# Changes in sensorimotor network dynamics in resting-state recordings in Parkinson’s disease

**DOI:** 10.1093/braincomms/fcaf282

**Published:** 2025-07-23

**Authors:** Oliver Kohl, Chetan Gohil, Nahid Zokaei, Michele T M Hu, Anna C Nobre, Mark Woolrich, Andrew Quinn

**Affiliations:** Oxford Centre for Human Brain Activity (OHBA), Welcome Centre for Integrative Neuroimaging, Department of Psychiatry, University of Oxford, Oxford OX3 7JK, UK; Oxford Centre for Human Brain Activity (OHBA), Welcome Centre for Integrative Neuroimaging, Department of Psychiatry, University of Oxford, Oxford OX3 7JK, UK; Oxford Centre for Human Brain Activity (OHBA), Welcome Centre for Integrative Neuroimaging, Department of Psychiatry, University of Oxford, Oxford OX3 7JK, UK; Department of Experimental Psychology, University of Oxford, Oxford OX2 6GG, UK; Division of Neurology, Nuffield Department of Clinical Neurosciences, University of Oxford, Oxford OX3 7JX, UK; Oxford Centre for Human Brain Activity (OHBA), Welcome Centre for Integrative Neuroimaging, Department of Psychiatry, University of Oxford, Oxford OX3 7JK, UK; Department of Experimental Psychology, University of Oxford, Oxford OX2 6GG, UK; Wu Tsai Institute and Department of Psychology, Yale University, New Haven, CT 06519, USA; Oxford Centre for Human Brain Activity (OHBA), Welcome Centre for Integrative Neuroimaging, Department of Psychiatry, University of Oxford, Oxford OX3 7JK, UK; Oxford Centre for Human Brain Activity (OHBA), Welcome Centre for Integrative Neuroimaging, Department of Psychiatry, University of Oxford, Oxford OX3 7JK, UK; Centre for Human Brain Health, School of Psychology, University of Birmingham, Birmingham B15 2TT, UK

**Keywords:** Parkinson’s disease, beta bursts, hidden Markov model, magnetoencephalography, sensorimotor network

## Abstract

Non-invasive recordings of magnetoencephalography have been used for developing biomarkers for neural changes associated with Parkinson’s disease that can be measured across the entire course of the disease. These studies, however, have yielded inconsistent findings. Here, we investigated whether analysing motor cortical activity within the context of large-scale brain network activity provides a more sensitive marker of changes in Parkinson’s disease using magnetoencephalography. We extracted motor cortical beta power and beta bursts from resting-state magnetoencephalography scans of patients with Parkinson’s disease (*N* = 28) and well-matched healthy controls (*N* = 36). To situate beta bursts in their brain network contexts, we used a time-delay-embedded hidden Markov model to extract brain network activity and investigated co-occurrence patterns between brain networks and beta bursts. Parkinson’s disease was associated with decreased beta power in motor cortical power spectra, but no significant differences in motor cortical beta-burst dynamics occurred when using a conventional beta-burst analysis. Dynamics of a large-scale sensorimotor network extracted with the time-delay-embedded hidden Markov model approach revealed significant decreases in the occurrence of this network with Parkinson’s disease. By comparing conventional burst and time-delay-embedded hidden Markov model state occurrences, we observed that motor beta bursts occurred during both sensorimotor and non-sensorimotor network activations. When using the large-scale network information provided by the time-delay-embedded hidden Markov model to focus on bursts that were active during sensorimotor network activations, significant decreases in burst dynamics could be observed in patients with Parkinson’s disease. In conclusion, our findings suggest that decreased motor cortical beta power in Parkinson’s disease is prominently associated with changes in sensorimotor network dynamics using magnetoencephalography. Thus, investigating large-scale networks or considering the large-scale network context of motor cortical activations may be crucial for identifying alterations in the sensorimotor network that are prevalent in Parkinson’s disease and might help resolve contradicting findings in the literature.

## Teaser

Kohl *et al*. report that sensorimotor network dynamics extracted from magnetoencephalogram recordings can distinguish patients with Parkinson’s disease from controls and highlight the importance of considering large-scale network context of motor cortical activations for understanding pathophysiological changes in Parkinson’s disease.

## Graphical Abstract

**Graphical Abstract:**
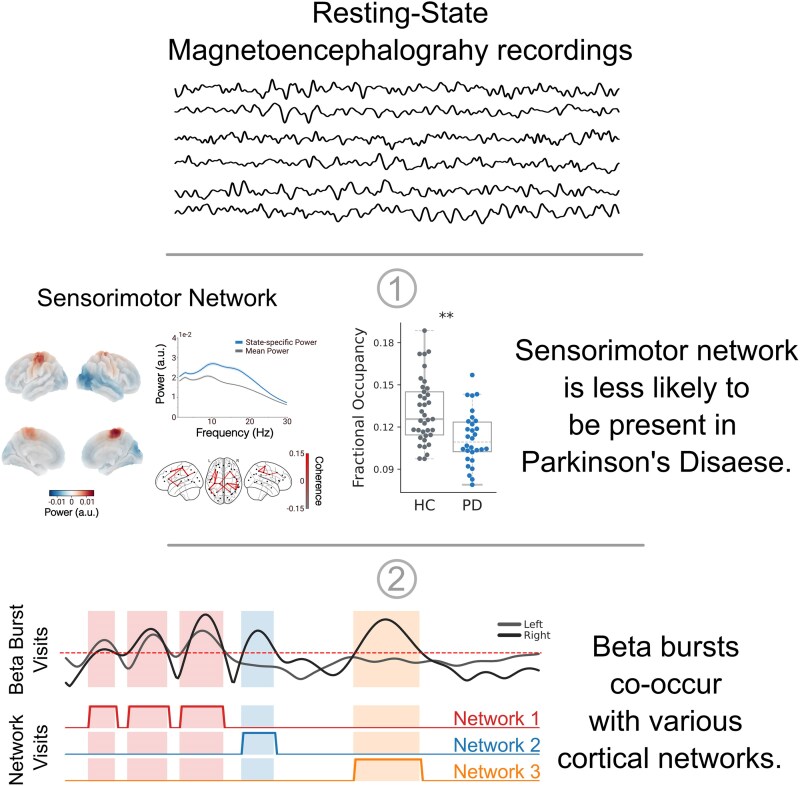

## Introduction

Parkinson’s disease (PD) is the fastest-growing cause of neurological-related disability worldwide.^1^ Its pathological hallmark is the progressive loss of dopaminergic neurons and accumulation of Lewy bodies in the substantia nigra pars compacta, compromising dopaminergic regulatory processes of the basal ganglia,^2,3^ and has been linked to motor impairments.^4^

An important effort is to define clear, sensitive and selective non-invasive biomarkers of PD pathology that can be measured across the complete duration of the disease in a wide range of patients. Magnetoencephalography (MEG) is a promising candidate because it directly measures magnetic fields arising from cortical neural activity with high temporal and good spatial resolution. This may enable the assessment of synaptic integrity and functioning in local and large-scale networks.^5^ Such non-invasive biomarkers should prove useful for the early identification of the disorder, treatment development and informing the search for biomarkers in low-density EEG recordings, which are more readily available in hospitals.

Experimental investigations using invasive methods in patients with progressed PD who underwent deep brain stimulation (DBS) surgery and animal models have advanced our understanding of the neurophysiological underpinnings of PD pathology. Excessive low beta oscillations in the basal ganglia-thalamocortical loop that are underpinned by prolonged periods of dynamically occurring low beta synchronization (=bursts) have been proposed to provide functional markers of PD.^4,6,7^ Observations of co-occurring subthalamic nucleus and motor cortical bursts,^8^ paired with reports of exaggerated motor cortical beta power in animal models^9,10^ and invasive motor cortical electrocorticogram recordings in patients with PD,^11,12^ pose the hypothesis that motor cortical beta power is exaggerated in patients with PD.

Interestingly, global observations of motor cortical beta power made in predominantly early stages of PD using non-invasive neurophysiological methods are inconsistent with the local observations in the invasive literature. For example, MEG and EEG studies that have investigated oscillatory changes in motor cortex have led to mixed findings. Some studies report no changes in motor cortical beta power,^13^ whereas others report decreased^14–17^ or increased beta power.^18,19^ Furthermore, the associations drawn between beta-power changes in motor cortex and motor symptom severity are mixed.^20,21^ Other reports show that PD-related decreases in the motor cortical power spectrum normalize following the administration of dopaminergic medication.^14,22,23^

Adopting a dynamic, burst-based approach has not proven sufficient to harmonize findings. Analyses of motor beta bursts in non-invasive MEG recordings have generated inconsistent findings regarding patterns of changes in their dynamic properties: decreases in motor cortical beta-burst rates,^24^ no group differences in burst rates but a steeper age-related decline in burst rates^25^ and increased burst durations and amplitudes in PD volunteers that normalized after DBS have been reported.^26^ Changes in burst rates were reported to scale with Bradykinesia symptom severity,^24,25^ and burst amplitudes were associated with Unified Parkinson Disease Rating Scale (UPDRS) hemi-body scores.^26^

This puzzle of inconsistent findings among the non-invasive studies, as well as between non-invasive and invasive studies, may be caused by many factors such as varying disease progression, symptomatology, assessment of intervention effects versus healthy control (HC) versus PD group contrasts, effects of surgery,^27^ or scale of measurements. Especially given the large scale of M/EEG measurements, it is likely that activity in a single scalp region constitutes different types of activity, with each type associated with different brain networks.

We propose that the large-scale network context within which motor cortical activations occur is a fundamental factor to consider when investigating non-invasively measured motor cortical activity. Beta activations recorded non-invasively from motor sensors likely include activity associated with brain networks beyond the sensorimotor network. For example, previous MEG studies demonstrated beta power decreases linked to spectral slowing in central and posterior cortical areas in PD.^28,29^ Importantly, in conventional spectral and burst analyses, a sensor or region of interest is pre-selected. However, the signals recorded from these regions indiscriminately reflect the summation across multiple active brain networks. We argue that to pinpoint pathological changes in PD accurately, it is essential to focus on motor cortical activations within the specific brain networks affected by the disorder. Consequently, methods such as time-delay-embedded hidden Markov models (TDE-HMM),^30^ which provide large-scale network descriptions of cortical brain activity, have the potential to dissect relevant motor cortical activity more effectively and thereby provide more precise markers of PD-related change. Considering the centrality and prevalence of motor symptoms in the diagnosis of PD, we hypothesize that more reliable markers of PD can be extracted by focusing on sensorimotor network occurrences and associated motor cortical beta activations.

In the present work, we investigate whether the large-scale network context of motor cortical activations helps identify pathophysiological changes in PD and, thus, is crucial for the identification of non-invasive biomarkers of PD. We test whether sensorimotor network dynamics are altered in patients with PD. We further hypothesize that motor beta bursts may be associated with various large-scale network contexts, not all of which are closely linked to pathological changes in PD. By focusing on sensorimotor network occurrences and motor cortical activations embedded within this network, it should be possible to tap into processes that are more closely related to the primary PD pathology.

We start by trying to replicate previously observed changes in static motor cortical oscillatory beta power and beta bursts extracted with amplitude thresholding.^24,26^ We then compare large-scale brain network dynamics extracted with a TDE-HMM between HCs and patients with PD to test whether previously observed decreases in burst rates^24^ and increases in burst amplitudes and durations^12,26^ can be witnessed in a sensorimotor large-scale network. Crucially, we finally investigate the co-occurrence patterns between large-scale networks and motor beta bursts to determine whether focusing on beta bursts associated with the sensorimotor network improves the discrimination between PD volunteers and HCs.

## Materials and methods

MEG recordings from 8-min resting-state scans with eyes open of 28 patients with PD and 36 HCs, matched in age and education, were analysed for this study (Table 1). The resting-state scans were collected alongside task-based data in two previous studies by our laboratory.^31,32^ The scanning protocol for the resting-state scans of both studies was matched. Before the scan, all participants were informed about the detrimental effects of movements on the quality of the brain activity recordings and were asked to sit as still as possible during the scan. MEG recordings were acquired at a sampling rate of 1000 Hz using a VectorView scanner. PD volunteers were asked to withdraw from their dopaminergic medication from 7 p.m. on the night before the MEG scan. Cognitive scores, measured with the Addenbrooke’s cognitive examination, did not differ significantly between the two groups in the included participants of the Zokaei *et al*.^32^ sample [*t*(28) = 0.42; *P* = 0.68]. For the Heideman *et al*. sample, significantly reduced Montreal cognitive assessment values in the normal to mild cognitive impairment range (26 ± 0.8) were reported for PD volunteers.^31^ PD volunteers were recruited via neurological clinics in Oxfordshire (UK) and the Dementias and Neurodegenerative Disease Research Network (DeNDRoN). An overview of the exclusion criteria of both studies can be found in Supplementary Table 1. Individual years since diagnosis, Addenbrooke’s cognitive examination or Montreal cognitive assessment scores, Hoehn and Yahr scores, UPDRS-III scores and Levodopa-equivalent daily dose are listed alongside demographic variables in Supplementary Table 2. Both studies were approved by the NHS Oxford Research Ethics Committee and the Research Ethics Committee of the University of Oxford and followed the Declaration of Helsinki. All participants gave written informed consent before participating.

**Table 1:** **Descriptive statistics of all participants included** ^a^

Demographics	HCs	PD
*N*	36	28
Sex (female/male)	13/23	15/13
Handedness (right/left)	34/2	25/3
Age (years), mean (range)	68.36 (60–80)	68.5 (54–83)
Education (years), mean (range)	15.36 (10–21)	14.68 (10–24)
Years since Diagnosis (years), mean (range)	N/A	2.86 (1–7)
UPDRS-III motor score, mean (range)	N/A	30.36 (11–55)
Hoehn and Yahr scale, mean (range)	N/A	1.77 (1–3)
Levodopa-equivalent daily dose (mg/day), mean (range)	N/A	365.80 (0–900)

^a^ *N*, number of participants; UPDRS, Unified Parkinson Disease Rating Scale.

### Software

All steps of the preprocessing were performed in MATLAB R2020a using the MATLAB version of the OHBA Software Library (OSL, https://github.com/OHBA-analysis/osl-core), Statistical Parametric Mapping (SPM12; https://www.fil.ion.ucl.ac.uk/spm/)^33^ or the HMM-MAR toolbox (https://github.com/OHBA-analysis/HMM-MAR).^34^ TDE-HMMs were run with the HMM-MAR toolbox.

**Figure 1:**
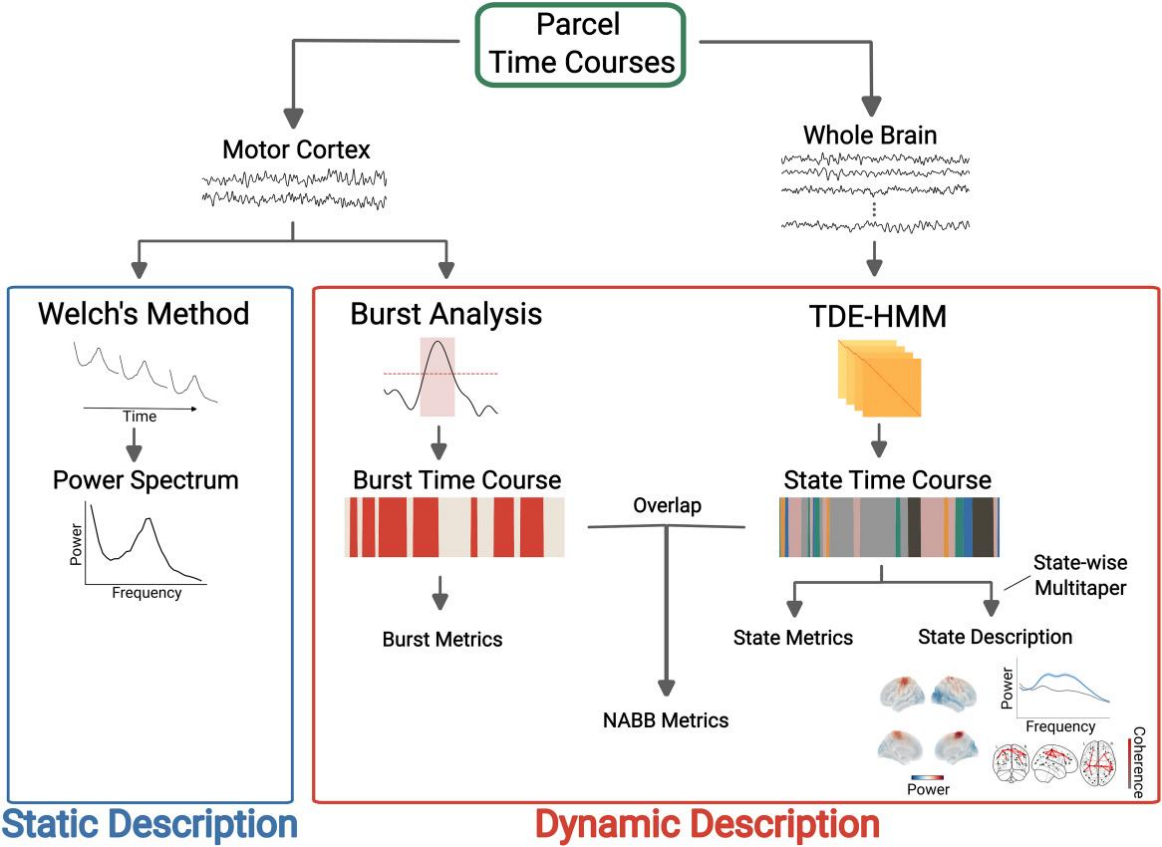
**Schematic of the different analyses reported in this paper.** All analyses were run on the parcellated and sign-flipped data. Welch’s method was applied to time courses of the left and right motor cortex to obtain a static description of motor cortical oscillations. The same time courses were used for the conventional amplitude-thresholding bursts analysis to extract motor cortical beta-burst time courses. From these burst time courses, burst metrics describing dynamic features of these events were calculated. A TDE-HMM was applied to the time courses of all parcels to extract HMM state time courses. Based on these state time courses, spectral state descriptions were calculated with a state-wise multi-taper approach, and state metrics were calculated to describe the dynamics of these states. Finally, conventional beta bursts were subdivided according to the HMM state with which they co-occurred, and metrics describing the dynamics of these NABBs were calculated.

Analyses of the TDE-HMM outputs were performed in Python 3.10. HMM state descriptions were estimated using OSL-Dynamics (https://github.com/OHBA-analysis/osl-dynamics).^35^ General linear models (GLMs) contrasting the two groups, while controlling for confounds, were calculated with GLMTOOLs (https://pypi.org/project/glmtools/), and cluster-based permutation tests were calculated as implemented in MNE-Python.^36^ Analysis scripts used for the analyses can be found here: https://github.com/OHBA-analysis/Kohl2023_PD_HMM. A schematic of all the analyses performed in this paper can be found in Fig. 1.

### Preprocessing

The MEG data were maxfiltered (Maxfilter 2.2) with the temporal extension option (–tsss) to attenuate components in the MEG signal originating from outside the head. The data were further transformed to a reference head position (–trans). Continuous information about the participants’ head position in the scanner, recorded with head position indicators, was utilized to correct for movements during the scan (–movecomp). The maxfiltered data were converted into SPM12 format in MATLAB for further processing. The MEG recordings were registered to the participants’ structural MRI scans with RHINO (Registration of Headshapes Including Nose). The data were downsampled to 400 Hz before applying a 0.1- to 125-Hz broadband filter and notch filters at 50 and 100 Hz using SPM12. Bad channels and bad segments were identified with osl_detect_artefacts. Across all participants, an average of four channels were rejected as bad channels and 5% of the recording duration was identified and removed as bad segments. To reject eye-movement and heartbeat artefacts in the data, OSL Independent Component Analysis (ICA) artefact removal was applied. ICA components highly correlated (≥0.5) with the electrooculogram or electrocardiogram channels were rejected. In cases where no subcomponents were linked to the artefact channels or more than four channels were rejected, manual artefact detection was conducted on the ICA output. On average, three ICA components (SD = ±1) per participant were removed through this procedure. The MEG sensor data were then reconstructed without the ‘artefactual’ components and carried forward to the source analysis.

Sensor normalization was applied to ensure equal contribution from MEGMAG and MEGPLANAR channels to the source localization.^37^ A linearly constrained minimum variance vector beamformer^37,38^ with a principal component analysis regularization rank of 50 projected MEG sensor data onto an 8-mm MNI152 standard brain template. Parcel-wise time courses were calculated for a weighted parcellation with 39 cortical regions.^39^ For each parcel, voxel contributions were weighted according to respective parcel weights and the first principal component across voxels was specified as the parcel’s time course.

For the HMM analysis, the source-reconstructed parcel time courses were broadband filtered to 1–45 Hz and downsampled to 250 Hz. We then performed symmetric multivariate leakage reduction, which corrects for both direct and inherited (so-called ghost interactions)^40^ in spatial leakage.^39^ Sign-flipping^34^ was applied next. Finally, the data were split into 10-s segments, and generalized extreme studentized deviate outlier detection^41^ was used to remove segments with exaggerated variance or kurtosis values. On average, 5% (SD = ±3%) of the data were identified as bad segments. These preprocessing steps ensured that the TDE-HMM analysis could accurately identify brain states without being influenced by outlier events. The resulting data were used for all subsequent analyses.

### Static power

To explore the signal power across a range of frequencies, we used Welch's method to compute power spectra. The algorithm was applied to each participant's individually *z*-standardized parcel time course using a window length of 500 samples and a window overlap of 250 samples to obtain relative power per frequency bin. After averaging relative power spectral values across the two superior motor parcels for each participant, average power at each frequency bin was contrasted between the PD and control group with a *t*-test and cluster-based permutations.^36^ This analysis yielded a cluster with significantly altered oscillatory power between 16 and 25.5 Hz between the two groups. Based on the assumption that time-averaged beta power, beta bursts and sensorimotor network activations provide different perspectives on the same underlying phenomenon, we used this cluster’s frequency range to inform the frequency band selection in subsequent burst analyses and burst- and state-specific beta power analyses.

### Burst analysis

We applied a conventional amplitude-thresholding burst analysis to the two superior motor parcels to explore the dynamics of individual beta-power events underpinning spectral power in the time domain. For each participant, the preprocessed data were filtered between 18 and 25 Hz based on the significant cluster obtained from the static motor cortical power group contrast. Next, we calculated amplitude envelopes of the time courses using the Hilbert transform and thresholded them at the 75th percentile separately for each participant. Finally, bursts were identified as instances in which the threshold was crossed for longer than one cycle of the lowest frequency (18 Hz) in the frequency range of interest.^42^ This resulted in binary on-versus-off time courses. A bilateral motor cortical on-versus-off burst time course was created by assuming a burst occurrence at instances when a burst occurred in at least one of the motor parcels. The average burst overlap between motor cortical parcels was 69% (SD = ±1%). Burst time courses of both motor cortical parcels were combined to enable fair comparisons with TDE-HMM states, which were predominantly bilateral. Using the combined burst on-versus-off time course, we extracted the fractional occupancy (FO, fraction of total time with bursts present), burst lifetimes (duration of burst occurrences) and state rates (number of burst occurrences per second). Additionally, we estimated motor cortical beta-power changes during bursts using a multi-taper approach.^34^ After subtracting the mean power across burst-on and burst-off periods, motor cortical beta-power estimates were averaged across both motor parcels to obtain beta-power changes during burst events.

### Time-delay-embedded hidden Markov model

#### TDE-HMM description

We employed a TDE-HMM on our data to investigate whether large-scale network information can be used to improve differentiation between the HC and PD groups. TDE-HMMs extract dynamic brain states in a data-driven manner, with each state representing a large-scale functional network characterized by distinct multi-region oscillatory activity, i.e. distinct power covariations and phase synchrony patterns.^30^ Compared to conventional single-region beta-burst analyses, the HMM provides a richer description by linking bursting activity in any region to the associated large-scale, multi-region network activity. In other words, each time a large-scale network occurs, it can be thought of as a transient bursting event of a large-scale, oscillatory network. The data-driven (unsupervised learning) nature of TDE-HMMs further obviates the necessity of setting *a priori* assumptions (i.e. region of interest, filter range, or beta-amplitude threshold) and thus reduces arbitrariness.

A detailed description of the inference process can be found elsewhere.^30^ In short, the standardized time series is described as a sequence of a defined number of hidden states in which only one state is active at any moment. Each state is defined by an observation model that captures a distinct auto- and cross-covariance pattern across regions. Autocovariance patterns are calculated for each sample by embedding it into a time window spanning ±7 samples and subsequently reducing its dimensionality by selecting a subset of principal components (2 × number of parcels) inferred with a principal component analysis. The dimensionality reduction reduces computational demands and prevents overfitting. To further reduce computational costs, we employed a stochastic variational inference approach.^43^ Importantly, the distinct auto- and cross-covariance patterns for each HMM state can capture different oscillatory activities, including distinct patterns of multi-region spectral power and phase locking.

#### State-specific power and coherence

State-specific power and coherence were calculated for each participant *post hoc* using the source-reconstructed data and the a-posterior probability time course of the states inferred by the TDE-HMM. Following Vidaurre *et al.,*^34^ a multi-taper applying seven Slepian tapers with a window length of 2 s was used to extract time-resolved power and coherence estimates between 1 and 45 Hz with a frequency resolution of 0.5 Hz. Time course data were weighted by the posterior state probabilities before applying the multi-taper, resulting in state-specific power and cross-spectral densities. To visualize large-scale network connectivity patterns, state-specific coherence values were thresholded at the 97th percentile to emphasize the most prominent connections depicted in the respective figures.

#### State metrics

Temporal metrics were computed to compare large-scale network dynamics between the two groups. These metrics were calculated for each participant using the padded a-posterior probability time courses of the states. The following state dynamics were extracted: FO (fraction of total time a state was present), lifetimes (duration of visits to a specific state), interval times (periods between visits to the same state), state rates (number of state visits per second) and motor cortical beta-power change during state visits. State-specific motor cortical beta-power change was obtained by first subtracting the mean power across all states from state-specific power spectra and then averaging the 16–25.5 Hz power in the superior motor parcels.

#### Robustness check

The number of states extracted by the HMM is a parameter that must be specified before running the HMM and can yield varying results. Since the inference of the algorithm is randomly initialized, different runs of an HMM with the same parameter settings on the same data can yield slightly different results. However, the main state features, such as state topologies and dynamics, as well as key findings of the analysis should be robust across different HMM runs with the same parameter settings and varying numbers of states. To ensure that the results obtained were robust, we ran 10 HMMs inferring 8, 10, and 12 states, respectively (Supplementary Fig. 1). The robustness of key findings was checked across all 30 HMMs. The representative results shown in the main figures come from an HMM with the lowest variational free energy (an approximation to the Bayesian model evidence) of all HMMs that looked to infer eight states.

### Motor beta bursts in large-scale network context

#### TDE-HMM state and burst overlap

To investigate the temporal overlap between conventionally estimated motor beta bursts and the occurrence of TDE-HMM network states, we calculated the percentage of samples where HMM state occurrences overlapped with motor beta-burst occurrences. This was achieved by dividing the sum of overlapping samples by the total number of burst samples, allowing for interpretation of the overlap as the percentage of the overall motor cortex beta-burst occurrences. The significance of the overlap between TDE-HMM states and motor beta-burst occurrences was calculated with non-parametric permutation tests. Empirical TDE-HMM state time courses were randomly shifted for each participant to generate null TDE-HMM state time courses that only differed in their temporal relationship to the empirical state time courses. Overlaps between the motor beta burst on-versus-off time courses and the shifted TDE-HMM state time courses were calculated as described above. Overlaps of motor beta bursts with the shifted TDE-HMM state time courses were contrasted against overlaps of bursts with the inferred TDE-HMM state time course with a directed *t*-test per TDE-HMM state. Bonferroni correction was applied to correct for multiple comparisons across states.

#### Calculation of network-associated beta-burst metrics

We segmented conventional motor beta bursts according to co-occurring large-scale networks derived in the TDE-HMM analysis to investigate whether focusing on motor beta bursts associated with sensorimotor network activations recovered significant group differences in burst dynamics. We refer to these segmented motor beta bursts as network-associated beta bursts (NABBs). Metrics describing NABB dynamics were calculated in a similar way to the TDE-HMM state metrics. To test whether group differences between PD volunteers and HCs only occurred for sensorimotor NABBs, we identified bursts overlapping with large-scale networks other than the sensorimotor network (other NABBs) and compared their FOs between the two groups. We further calculated NABB metrics for each large-scale network and compared them between the two groups.

### Statistical analyses

Group contrasts for burst, network-state and NABB metrics were assessed using GLMs with group constants. We included the dataset (Heideman *et al*. versus Zokaei *et al*.), age, sex, education and handedness as regressors to control for potential confounds (Supplementary Fig. 2). Significance was assessed using maximum *t*-statistic permutation tests by shuffling group labels before calculating the above-mentioned GLMs 10 000 times. For group contrasts of large-scale network and NABB metrics, where a separate contrast is performed for each network, maximum *t*-statistic pooling across networks was used to determine significance while controlling for multiple comparisons.^44^ For each permutation, the largest *t*-statistic across all states was selected to construct a single null distribution, which was used to determine the significance of the metric’s group contrast for each state. Observed *t*-statistics of state or NABB metrics were deemed to be significant if they exceeded the *P* < 0.05 threshold obtained from the null distribution.

UPDRS motor scale items were combined into Bradykinesia/Rigidity and Tremor scores.^4,45^ Relationships between metrics and UPDRS scores were assessed with separate GLMs, one for each of the metrics that significantly differed between the two groups. In these models, metric scores were predicted from Bradykinesia/Rigidity and Tremor scores while controlling for the above-mentioned confounds (Supplementary Fig. 3). Maximum *t*-statistic permutation tests with 10 000 permutations were applied to determine the significance of the associations. For time-averaged beta power, which could be calculated separately for both hemispheres, this analysis was performed with UPDRS scores from the more impaired body side and beta power obtained from the contralateral superior sensorimotor cortex.

## Results

### Decreased static beta power in PD but no differences in conventional beta-burst analysis

**Figure 2:**
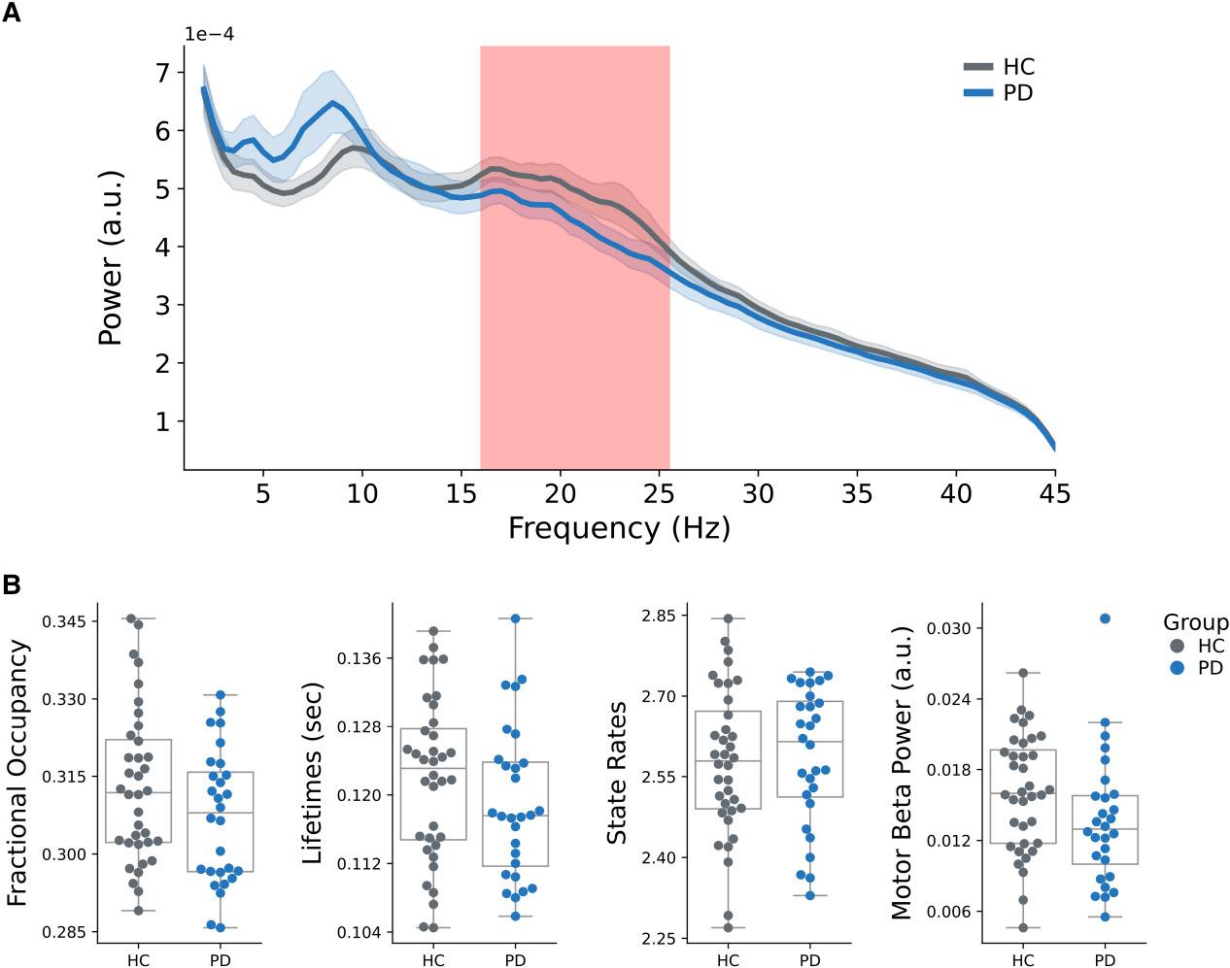
**Motor Cortical Beta Oscillatory power decreased in patients with PD in the static power spectrum, but there were no changes in conventional amplitude-thresholding beta-burst analysis.** (**A**) Motor cortical average power spectra of HC and PD groups. The cluster with significant power differences [peak frequency = 22.6 Hz; mean *t*(57) = 2.65, *P* = 0.03], obtained from the cluster-based permutation test, is marked in red. (**B**) GLMs comparing 16–25.5 Hz motor cortex beta-burst metrics between the HC and PD group did not reveal significant group differences (all *P* > 0.21). Note that the 16–25.5 Hz frequency range was selected based on the significant cluster in power-spectrum analysis.

The group comparison of motor cortical mean static power spectra (i.e. averaged over the whole resting-state scan) between the HC and PD group (Fig. 2A) yielded a significant cluster in the beta range (16–25.5 Hz) with decreased power in the PD group [peak frequency = 22.6 Hz; mean *t*(57) = 2.65, *P* = 0.03]. Another non-significant 3.5–8.5-Hz cluster with increased power in PD volunteers was found as well [peak frequency = 8 Hz; mean *t*(57) = 2.78, *P* = 0.06]. Beta power averaged across the range of the significant cluster was not associated with Bradykinesia/Rigidity [*t*(13) = 0.84, *P* = 0.42] or Tremor [*t*(13) = −0.61, *P* = 0.55] scores of the participants’ more impaired body sides.

Comparisons of conventional motor cortical beta-burst metrics between the HC and PD groups (Fig. 2B) did not reveal significant changes in metrics describing the dynamic properties of bursts (all *P* > 0.21).

### HMMs describe fast, transient network dynamics

**Figure 3:**
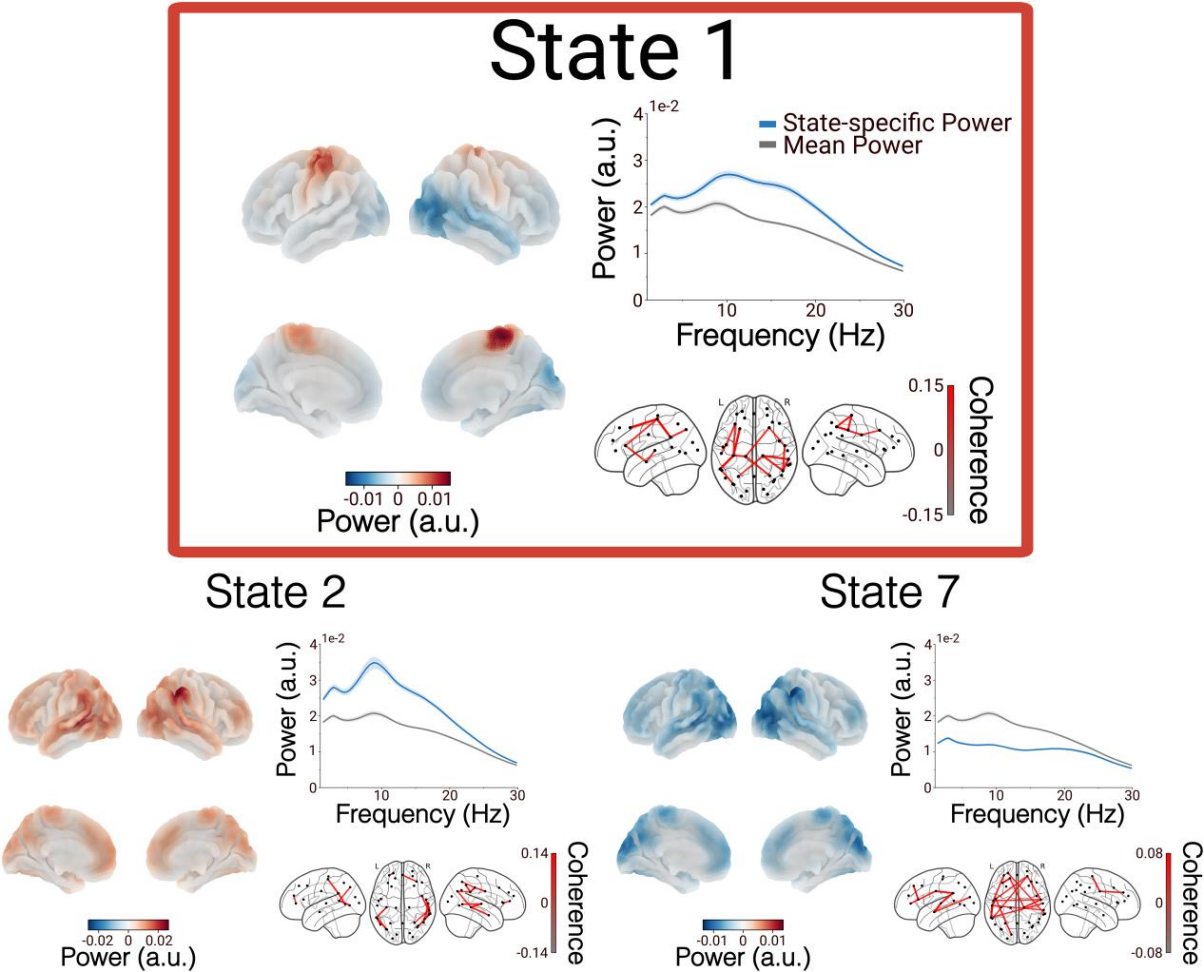
**Dynamically switching large-scale networks inferred with TDE-HMM.** A subset of HMM states relevant to this study is shown. State 1, corresponding to the sensorimotor network, is highlighted in red. Each state is depicted using three panels that present averaged data across all participants. Left: Spatial maps of oscillatory power (2–30 Hz), shown relative to the mean power across all states, projected onto cortical surfaces. Top right: State-specific motor cortical power spectra (blue) compared to the time-averaged power spectrum across all states (grey). Bottom right: Coherence networks (2–20 Hz) thresholded at the 97th percentile to highlight prominent connections. A full overview of all eight states inferred using the TDE-HMM is provided in Supplementary Fig. 4.

Descriptions of temporally and spectrally resolved large-scale networks inferred by the TDE-HMM are depicted in Fig. 3. Each time a state switches ‘on’, it can be thought of as a transient bursting event of distinct oscillatory network activity. These networks exhibited power covariance and phase connectivity patterns consistent with previous studies extracting large-scale networks from MEG resting-state data.^30,46^ Importantly, State 1 occurrences were linked to increased wideband power and coherence in motor cortical areas. These wideband power increases showed strong spatial correlations with well-established sensorimotor network activation maps from the neuroimaging literature (Supplementary Fig. 5).^47^ Therefore, from here on, we will refer to State 1 as the sensorimotor state. Since changes in motor cortical dynamics have been reported in PD,^12,24,26^ we will focus on the sensorimotor state in the following analyses.

The TDE-HMM state metrics (Supplementary Fig. 6) provide a summary of the temporal dynamics of the large-scale networks captured by the TDE-HMM. Extracted networks had similar mean FOs, ranging between 11 and 16%. FO values across all states and participants were smaller than 47% and larger than 3%, indicating that the brain networks mix well within participants, i.e. all participants’ data were not represented by a single state, and all networks were visited for each participant. Mean lifetimes varied between 50 and 90 ms and interval times between 460 and 778 ms, which is in line with previous studies.^30,46^

### Fast, transient network dynamics relate to individual differences in static spectral power

**Figure 4:**
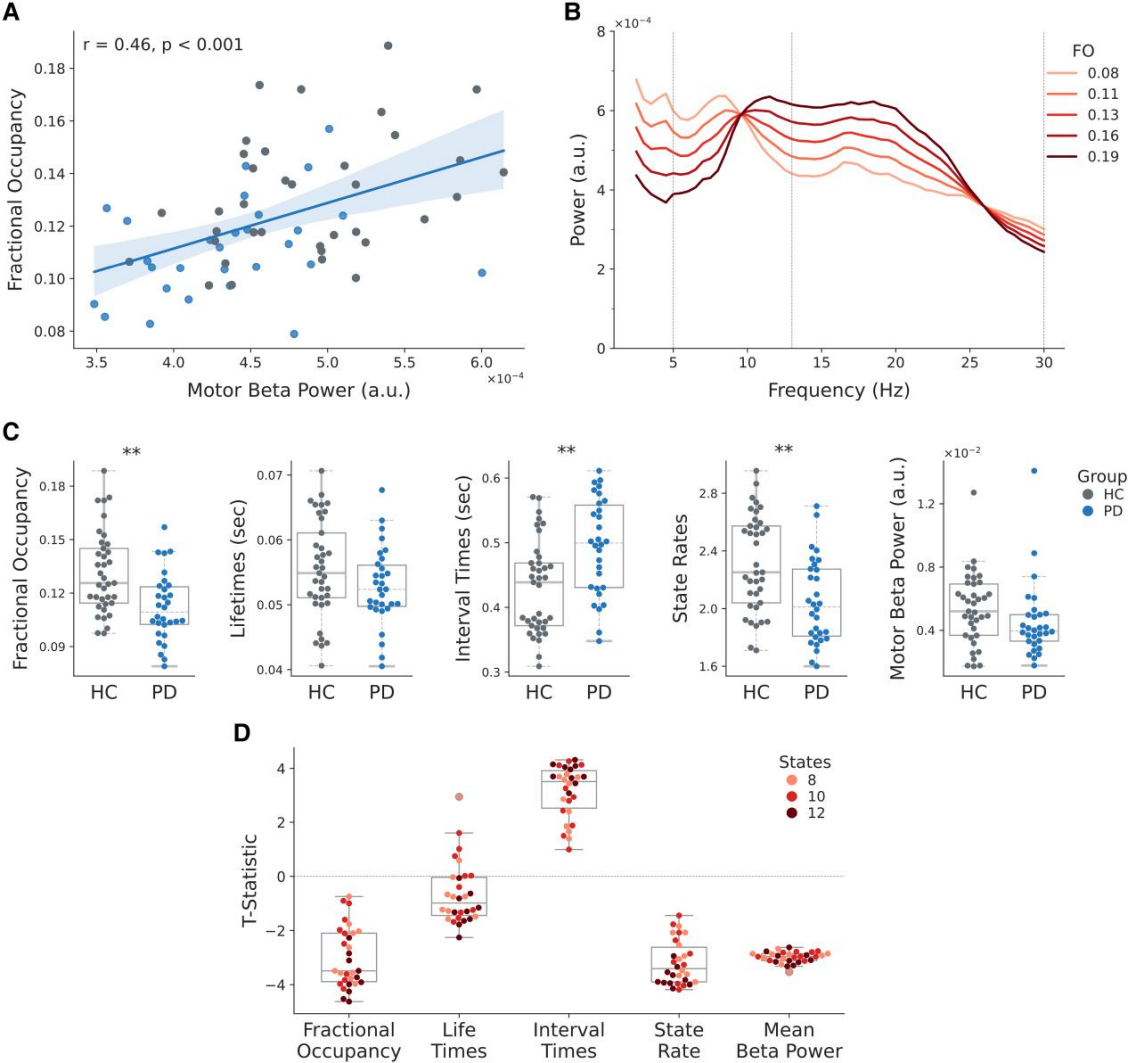
**Sensorimotor network dynamics are associated with motor cortical beta power and differ significantly between the HC and PD groups.** (**A**) The significant Pearson’s correlation between each participant’s FO of the sensorimotor state and motor cortical beta power confirms that brain state metrics relate back to static oscillatory activity. Each dot represents the values of one participant, and the colour indicates the participant’s group, with HCs coloured in grey and patients with PD in blue. (**B**) Projections of a GLM that predicts how spectral power in the motor cortex varies over subjects, using the FO of the sensorimotor state for each subject. Note that a different GLM was fitted for each frequency. This indicates that as the rate of occurrence (i.e. the FO) of the sensorimotor network increases, so does the alpha and beta power in motor areas. (**C**) Group contrasts of FOs [*t*(57) = −3.97, *P* < 0.01], mean lifetimes [*t*(57) = −1.58, *P* = 0.5], mean interval times [*t*(57) = 3.84, *P* < 0.01], state rates [*t*(57) = −3.69, *P* < 0.01] and motor beta power [*t*(57) = −2.12, *P* = 0.16] of the sensorimotor network are presented (HCs = grey; PD = blue). The significance of group differences is assessed with maximum *t*-statistic permutation tests controlling for multiple comparisons across states. *t*-statistics were calculated from GLMs accounting for confounds. (**D**) *t*-statistics of significant group differences in state metrics inferred with the TDE-HMM model with 8 states and lowest free energy are robust across 30 model fits extracting the 8, 10, or 12 states. Each dot represents the *t*-statistic of a GLM contrasting the respective state metric from one of the fitted TDE-HMMs. **P < 0.01; *P < 0.05.

We next assessed whether brain network probabilities related to static spectral power by correlating FOs of the sensorimotor network with motor cortical beta power across participants of both groups combined (Fig. 4A). As expected, increased occurrence (i.e. FO) of the sensorimotor network was associated with increased beta power, indicating its contribution to the observed power variations across participants. To estimate how network occurrences influence power across a wide range of frequencies, GLMs predicting individual participants’ power spectra from the sensorimotor network probabilities were fitted, and predictions of the obtained models for varying network FOs were extracted (Fig. 4B). Note that a different GLM was fitted for each frequency. Predictions indicated that participants with higher FO of the sensorimotor state demonstrate increased alpha and beta power in motor cortical areas.

### Dynamics of sensorimotor network are associated with diagnostic group but not symptom severity

Group comparisons of large-scale network dynamics revealed that the sensorimotor network occurred less frequently in PD volunteers. Contrasts between state metrics of the HC and PD groups (Fig. 4C) indicated that the FOs [*t*(57) = −3.97, *P* < 0.01] and state rates [*t*(57) = −3.69, *P* < 0.01] of the sensorimotor state were significantly decreased, whereas interval times were significantly increased [*t*(57) = 3.84, *P* < 0.01], in the PD group. Notably, higher FOs of the sensorimotor state were associated with higher motor cortical beta power (Fig. 4A and B). Consequently, significant reductions in FOs in the sensorimotor network in PD are in line with reduced static motor cortical beta power in PD. Additionally, increased lifetimes of a right temporal network (State 8) were observed in PD [*t*(57) = 2.78, *P* = 0.04]. Group comparisons of all other state metric–brain state combinations did not reach significance (all *P* > 0.06) (Supplementary Fig. 7). In all these analyses, we corrected for multiple comparisons across states for each metric separately with maximum *t*-statistic permutation tests. Repeating this analysis for 29 other HMMs extracting 8, 10, or 12 states demonstrated that the observed group differences in the sensorimotor network were robust across different HMM inferences and number of states (Fig. 4D; Supplementary Fig. 8).

Correlations between sensorimotor state metrics, which differed significantly between the HC and PD groups, and motor symptom severity scores were assessed with GLMs accounting for potential confounds. No significant associations between sensorimotor state metrics of PD volunteers and Bradykinesia/Rigidity (all *P* > 0.27) or Tremor scores (all *P* > 0.56) were observed (Supplementary Fig. 9).

### Motor cortex beta bursts are associated with multiple large-scale networks

**Figure 5:**
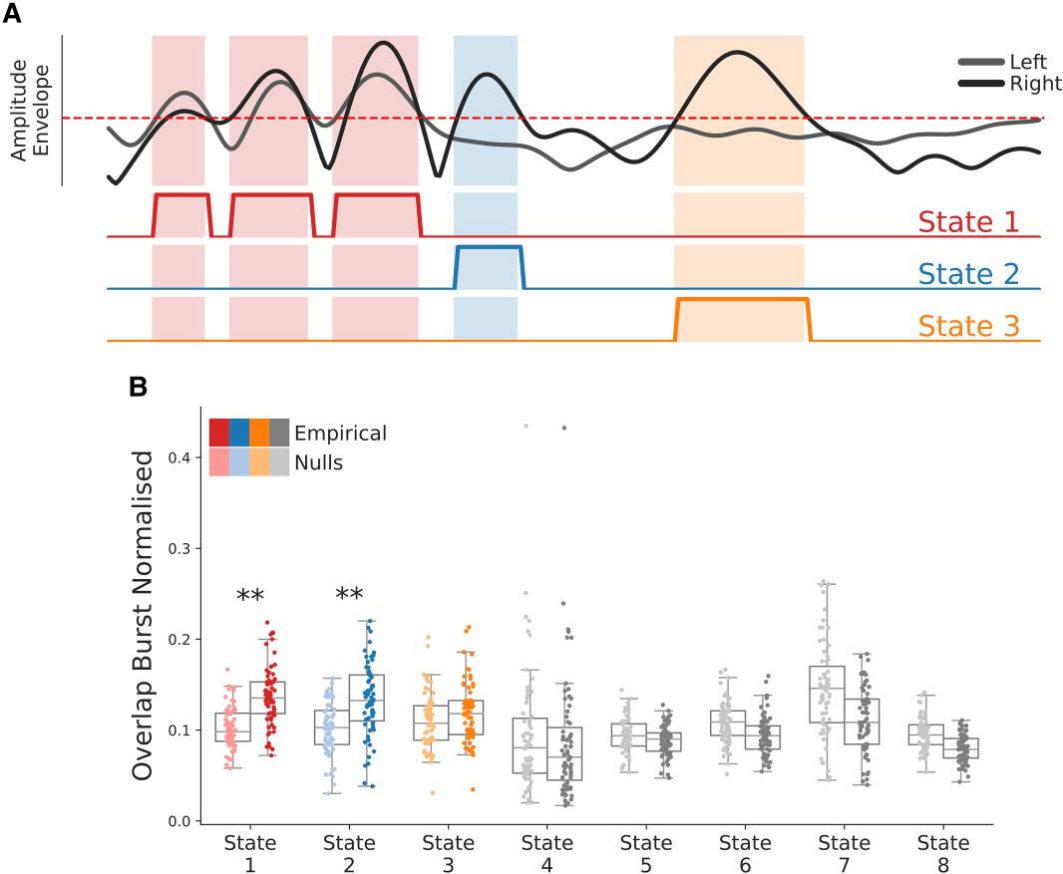
**Motor cortex beta bursts extracted with amplitude-envelope thresholding approach do not solely co-occur with sensorimotor network activations.** (**A**) Schematic depicting how the overlap between the occurrence of conventional beta bursts and HMM network state visits is calculated. Amplitude envelopes of the left (grey) and right (black) motor cortex are thresholded using the 75th percentile (dashed red line). HMM state on-versus-off time courses are depicted as red, blue and yellow lines below. Shaded areas indicate periods in which amplitude envelopes cross the 75th percentile and HMM state occurrences overlap. (**B**) Beta bursts showed significant overlap with both the sensorimotor network [*t*(132) = 7.47, *P* < 0.001] and State 2 [*t*(132) = 5.44, *P* < 0.001]. For each participant, the average temporal overlap between conventional beta bursts and each HMM state's occurrences was computed and is shown as an individual dot in strong colours. To create a null distribution, HMM state time courses of all participants were randomly shifted to disrupt temporal alignment, and overlaps were recomputed. Each of these null overlaps is displayed as a single dot in muted colours. The significance of the observed overlaps of all participants was tested against these null overlaps using directed *t*-tests. Overlaps between beta bursts and HMM states linked to decreases in motor cortical beta power are shown in grey. Bonferroni correction was applied to account for multiple comparisons across states. **P < 0.05; *P < 0.01 after Bonferroni correction.

We next compared the TDE-HMM network state time courses and conventional motor beta burst on-versus-off time courses (Fig. 5). This revealed that the occurrence of beta bursts overlapped with visits to the sensorimotor state [*t*(132) = 7.47; *P* < 0.001]. This indicated that both analyses were sensitive to similar events in the motor cortex. However, motor beta-burst occurrences also significantly overlapped with State 2 [*t*(132) = 5.44, *P* < 0.001], which is a state linked to widespread, increased broadband power (Fig. 5B). This suggested that while motor cortex beta bursts can be associated with the sensorimotor network, they can also be associated with other networks.

### Sensorimotor NABB dynamics are sensitive to PD

We found that conventional beta bursts in the motor cortex were not predictive of PD. At the same time, we observed that beta bursts can be associated (through overlapping co-occurrence) with multiple networks and not just the sensorimotor network. To investigate whether focusing on motor beta bursts associated with sensorimotor network activations reveals significant group differences in burst dynamics, we segmented conventional motor beta bursts according to co-occurring HMM networks into NABBs. For example, in the schematic in Fig. 5A, the overlaps indicate that the first three beta bursts are State 1 NABBs, the next burst is a State 2 NABB and the final burst is a State 3 NABB.

**Figure 6:**
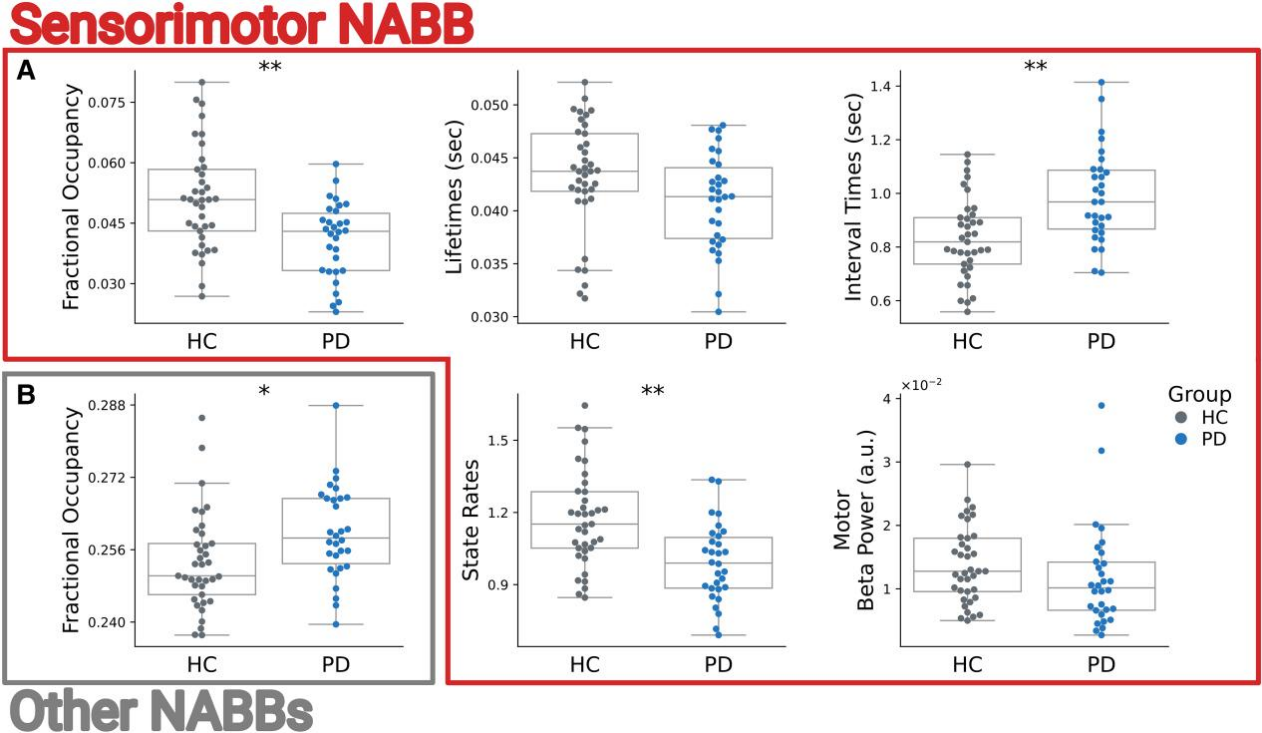
**State metrics of sensorimotor and frontal NABB dynamics significantly differ between the HC and PD groups.** (**A**) Group contrasts of sensorimotor NABB FOs [*t*(57) = −3.98, *P* = 0.002], mean lifetimes [*t*(57) = −2.21, *P* = 0.17], mean interval times [*t*(57) = 4.33, *P* < 0.001], state rates [*t*(57) = −4.41, *P* < 0.001] and motor beta-power [*t*(57) = −1.9, *P* = 0.19] are depicted (HC = grey; PD = blue). (**B**) FOs of occurrences of all but the sensorimotor NABBs were significantly increased in PD volunteers [*t*(57) = 2.39, *P* = 0.03]. Significance of group differences is assessed with maximum *t*-statistic permutation tests controlling for multiple comparisons across states. *t*-statistics were calculated from GLMs accounting for confounds. **P < 0.01; *P < 0.05.

We found group differences in FO [*t*(57) = −3.98, *P* = 0.002], interval times [*t*(57) = 4.33, *P* < 0.001] and state rates [*t*(57) = −4.41, *P* < 0.001] for sensorimotor NABBs (Fig. 6A; Supplementary Fig. 10). This is similar to what we found with the pure TDE-HMM analysis in Fig. 4. In short, by titrating the conventional motor cortex beta bursts according to their co-occurrence with large-scale networks, we recovered the group differences observed in the pure TDE-HMM analysis.

To further test whether PD-related decreases in sensorimotor NABBs were specific to the sensorimotor network, we calculated the probability of bursts to overlap with any of the other networks (other NABBs) for the two groups. We observed that these other NABBs were significantly more likely to happen in the PD group [*t*(57) = 2.39, *P* = 0.03; Fig. 6B]. This suggests that the decrease in sensorimotor NABBs may be attenuated by activity in other NABBs in conventional burst analyses that ignore the network context of motor beta bursts. In follow-up analyses investigating whether certain NABBs drive the PD-associated significant increase in the other NABBs analysis, we found only State 7 NABBs to be significantly more likely [*t*(57) = 2.95, *P* = 0.04] and to show longer lifetimes in PD volunteers [*t*(57) = 3.08, *P* = 0.02] (Supplementary Figs. 11 and 12). Strikingly, State 7 NABB FO was significantly associated with motor cortical-cortical high beta-to-gamma coherence [*r*(62) = 0.39, *P* = 0.002] and Bradykinesia/Rigidity scores [*t*(20) = 2.14, *P* = 0.04] (Supplementary Figs. 13 and 14), implying that State 7 NABBs identify moments of motor cortical-cortical hyperconnectivity that scale with motor symptom severity. All other NABBs did not significantly differ between the two groups.

## Discussion

In the present study, we investigated whether analysing motor cortical activations within their large-scale brain network contexts provides a more sensitive measure of motor cortical oscillatory changes in patients with PD. The static power-spectrum approach showed decreases in motor cortical beta power in the PD group. Conventionally estimated motor beta-burst dynamics did not differ between the two groups, whereas large-scale network dynamics obtained with a TDE-HMM revealed decreases in the probability of the sensorimotor large-scale network being present. We hypothesized that the differing findings may be due to different levels of network specificity of these two approaches. Accordingly, we demonstrated that conventional motor cortex beta bursts did not solely co-occur with activation of the sensorimotor network but also with more widespread network activations. Separating motor cortex beta bursts co-occurring with sensorimotor network activations from bursts co-occurring with activations of other large-scale networks allowed us to recover significant group differences between the HC and PD group in burst visits, similar to what was observed in the pure large-scale network analysis. Importantly, group differences between beta bursts co-occurring with sensorimotor network visits and beta bursts co-occurring with other large-scale networks were in opposing directions, suggesting that these opposing changes may diminish group contrasts in conventional burst analyses that ignore the network context of motor cortical activations. Taken together, our findings highlighted the importance of adopting a large-scale network perspective to describe changes in cortical oscillatory activity in PD accurately and point to an important source of inconsistencies in the non-invasive literature.

### Static motor cortex-focused analysis reveals changes in oscillatory activity

The static power-spectrum analysis revealed significant decreases in motor cortical beta power. In line with this, decreased motor cortical beta power in PD^14,16^ and increased beta power after administration of dopaminergic medication^14,22,23^ have been previously reported. Heinrichs-Graham *et al*.^14^ proposed that neurodegeneration in the substantia nigra pars compacta disinhibits inhibitory projections from the globus pallidus interna and substantia nigra pars reticulata to the thalamus, which in turn reduces its excitatory projections to motor cortical areas, resulting in reductions of beta power.^14^ In line with this hypothesis, PD volunteers with stronger substantia nigra neurodegeneration have demonstrated decreased left sensorimotor beta power.^48^ However, it is important to stress that electrophysiological recordings of motor cortical beta oscillations have also yielded increases or no changes in motor cortical beta power.^20,49^ Additionally, widespread beta power decreases, potentially linked to spectral slowing, have previously been observed in central and posterior regions of the brain.^28,29^ Thus, it is likely that motor cortical beta power decreases do not only reflect pathological processes in the basal ganglia-thalamocortical loop but also widespread cortical alterations in beta oscillatory power. It remains an open question what causes varying findings on motor cortical beta changes in MEG recordings of PD volunteers. Methodological issues^12^ or systematic differences in PD samples across studies, i.e. differing medication status, PD subtypes, motor status or disease progressions,^20^ might be potential reasons. Another reason may be that the spatial extent^50^ or network context of motor cortical high beta-power events needs to be considered to dissect beta events that may be affected by PD in different ways.

### Sensorimotor large-scale network changes in the PD group

The dynamics of the sensorimotor network, extracted with the TDE-HMM analysis, indicated a lower probability of this network being present, reflecting decreases in the occurrences and longer intervals between visits. The observed reduction of sensorimotor network occurrences in PD volunteers replicates previous findings using static networks derived from ICA,^51^ network-based statistics,^52^ and motor cortical beta bursts.^24^ Burst rate reductions were reported to be significantly associated with Bradykinesia scores,^24^ whereas state probability changes observed in our TDE-HMM analysis were not significantly associated with motor symptom severity scores. In contrast to these findings, longer lifetimes and higher amplitudes of motor cortical beta bursts have been reported for patients in advanced PD disease stages who have undergone DBS surgery.^12,26^ Furthermore, we linked variations in the FOs of the sensorimotor network to variations in static beta power, suggesting that these changes in large-scale network dynamics underpin static power-spectrum measures. In sum, our findings indicate that large-scale network descriptions of brain activity enable distinguishing between PD volunteers and HCs and shed light on the dynamic underpinnings of group differences in the static power spectrum.

Importantly, extrapolating results from studies investigating the role of beta activity in the basal ganglia-thalamocortical loop through invasive recordings of PD patients, one would hypothesize that motor cortical beta power is increased in PD volunteers. This runs counter to various findings on motor cortical beta power changes reported in non-invasive M/EEG studies^20,21^ and to our specific observations of decreased beta power and sensorimotor network occurrence probability. Several factors could account for this surface-level inconsistency between invasive and M/EEG studies: (i) Volunteers in invasive electrocorticogram or DBS studies tend to have more severe disease progressions, symptomatology, exposure to and ‘wearing off’ of dopaminergic medication, and they may be less heterogeneous due to surgery qualification criteria. (ii) Comparisons of on versus off treatment, prevalent in invasive studies, are not equivalent to comparisons of PD versus HC groups, prevalent in non-invasive studies. (iii) DBS-electrode implantation has been reported to alter cortical network activity.^27^ (iv) Due to the larger scale of measurement, M/EEG may pick up changes in oscillatory power due to other PD pathology-related factors, e.g. oscillatory slowing, which dilutes basal ganglia-thalamocortical loop-related motor cortical activity in MEG recordings. Studies systematically investigating the effect of these factors on the inconsistencies between invasive and non-invasive studies may help improve our understanding of how the findings of these modalities relate to each other. This, in turn, would enable drawing better conclusions about PD-related pathologies that underpin non-invasive electrophysiological biomarkers.

### Network context identifies PD-related changes in motor cortical activations

In contrast to conventional amplitude-thresholding burst analyses, the spatiotemporal nature of large-scale networks allows for increased confidence about the spatial extent of network activation at each moment. We show that it is crucial to consider the underlying network distributions of transient, high-amplitude beta events when trying to identify sensorimotor network-specific markers of PD or other disorders. In contrast to the previous reports,^12,24–26^ we did not initially observe changes in motor burst metrics in PD volunteers. Instead, we demonstrated that our conventional amplitude-threshold beta-burst analysis did not only identify bursts co-occurring with sensorimotor network activity but also during occurrences of more widespread network activations. By separating motor beta bursts co-occurring with the sensorimotor network from motor beta bursts co-active with other large-scale networks, we revealed significantly decreased FOs and burst rates in motor beta bursts of PD volunteers, similar to what has been reported by Vinding *et al*.^24^ These findings suggest that a large-scale network perspective enables a more precise description of PD-related changes in sensorimotor network activity.

Motor beta bursts associated with different large-scale networks showed differing patterns of change. In contrast to sensorimotor NABBs, co-occurrence probabilities of beta bursts with all other large-scale networks (combined) were significantly increased in PD volunteers. This indicates that analysing dynamics of all motor cortex beta bursts together, without subdividing them according to co-occurring networks, attenuates group differences. Thus, it may be crucial to take a large-scale network perspective, considering spatiotemporal information, to extract sensorimotor network activity that reliably differentiates between HCs and patients with PD.

Taken together, our findings suggest that a large-scale network perspective is an important factor to consider in the search for non-invasive markers for PD. Future research investigating dynamic large-scale networks in samples with varying motor cortical beta power changes will allow for tests of whether these variations are underpinned by alterations in different large-scale networks. If this hypothesis holds, a dynamic large-scale network perspective may provide a framework unifying the inconsistent findings in the non-invasive literature and provide very strong candidates for non-invasive PD markers that can be measured throughout the whole course of the disease.

### Putative contributions of NABBs

Furthermore, investigating which of the non-sensorimotor NABBs is driving the increases in the PD group in the other NABBs analysis revealed that only State 7 NABBs were more likely to be present and had significantly longer lifetimes. Strikingly, the increase in lifetimes agrees with findings of increased burst lifetimes by Pauls *et al*.,^26^ whereas the decrease in sensorimotor NABB rates is in line with reports of decreased burst rates by Vinding *et al.*^24^ Thus, it is tempting to speculate that different beta bursts can be disentangled by considering simultaneously occurring large-scale networks. Differences in the activation of these NABBs in different PD subtypes may have the potential to reconcile differing findings in the literature.

Increased State 7 NABB lifetimes match burst dynamics expected based on invasive studies of the basal ganglia-thalamocortical loop.^8^ Thus, they may provide a dynamic window in which basal ganglia-thalamocortical loop-related activity can be picked up in M/EEG recordings of the motor cortex. Accordingly, State 7 NABB FO was the only extracted electrophysiological variable found to be significantly associated with motor symptom severity scores. If this hypothesis holds, basal ganglia-thalamocortical projections would not be associated with a state of increased beta oscillatory power in the sensorimotor network but rather with a state of motor cortical-cortical beta-to-gamma hyperconnectivity. In line with this, a cortico-cortical high beta-to-gamma network encompassing motor, occipitoparietal, middle temporal and prefrontal cortices has been reported to be modulated by DBS stimulation.^53^ This hypothesis should be treated carefully as this analysis was exploratory and requires replication in other datasets. Studies investigating the dynamics of subthalamic nucleus-motor cortical connectivity and its cortical network context could test this hypothesis and provide non-invasive markers for subcortical dysfunction that would enable tracking of the progression of basal ganglia dysfunction from the earliest to late stages.

### Limitations

The sensitivity of dynamic large-scale network markers to motor symptom severity may be compromised because the majority of extracted networks demonstrate bilateral activation patterns. In the context of PD, where the expression of motor symptoms is often asymmetric, this likely reduces the sensitivity to associations between neural measures and motor symptom severity scores.

Previous research on PD patients with moderate to severe motor symptom severity and resting tremor symptomatology demonstrated that beta power is reduced during resting tremor periods.^54,55^ PD volunteers in our sample demonstrated lower motor symptoms and tremor severity. There was also no significant association between beta power and rest tremor UPDRS-III scores, assessed immediately before the scan in the off-medication state. Nevertheless, we cannot rule out that resting tremors during the scan may have contributed to beta oscillatory power reductions. Future studies should aim to collect accelerometry data during MEG scans, allowing resting tremors to be quantified and accounted for during the scan.

The spatial resolution of the used parcellation is limited. All our analyses were conducted on source-localised data based on a weighted parcellation with 39 cortical regions.^39^ Since several motor areas, i.e. the supplementary motor area or motor cortex, are represented by the same parcels, no conclusions about different processes in these areas can be made. Refinement of the parcellation may allow for more precise claims about areas involved in the respective networks.

### Summary

In sum, the present study demonstrated that the spatiotemporal context of motor cortical activations is important for identifying PD-related changes in neuronal activity. We fitted a TDE-HMM to our data, providing a rich spatiotemporal description, which can be linked back to the fundamental static power spectrum. Sensorimotor network dynamics distinguished between patients with PD and HCs, whereas a conventional amplitude-thresholding burst analysis failed to do so. The nature of the TDE-HMM approach provides greater confidence in identifying sensorimotor network beta activity because of the full network dynamics model. Finally, we provided evidence that relating conventionally estimated motor beta bursts to large-scale network activity may be crucial for exploring PD-related changes in the sensorimotor network and may help clarify the contradicting findings in the literature. This work highlights the importance of taking a large-scale network perspective when attempting to identify non-invasive cortical biomarkers for PD.

## Supplementary Material

fcaf282_Supplementary_Data

## Data Availability

The conditions of our ethics approval do not permit public archiving of the data supporting this study. Sharing data requires a formal data-sharing agreement following ethics procedures governing the re-use of sensitive data. Readers seeking access to the data should contact the first author.
