# Supplementary material for: Changes in sensorimotor network dynamics in resting-state recordings in Parkinson’s disease
Source: Brain Commun. 2025 Jul 23;7(4):fcaf282. doi: 10.1093/braincomms/fcaf282 (PMC12318717; doi:10.1093/braincomms/fcaf282)
Supplement: fcaf282_Supplementary_Data [file fcaf282_supplementary_data.pdf]

Changes in sensorimotor network dynamics in resting-state Magnetoencephalogram recordings of Parkinson's Disease patients

Supplementary Material

Table of Contents

Detailed description of combined PD volunteer samples 3

Detailed description of demographic and clinical variables 4

Free Energy of different TDE-HMM fits 7

Supplementary Figures for GLM-Analyses 8

Summary of GLMs used for group comparisons between HCs and PD patients. 8

Summary of GLMs used to associate Variables with Motor Symptom Severity Scores. 9

TDE-HMM State Overview 10

Spatial correlations between TDE-HMM states and established sensorimotor network 12

HMM-State Metric Analysis 14

State Metrics across all participants 14

State Metric Group Contrast 15

HMM-State Metric Analyses Robustness 16

State Matching Procedure 16

Robustness of Group Contrast 17

Robustness of State Metric x UPDRS score association 18

Sensorimotor NAABs analysis robustness 20

NABB Metric Analysis 22

Robustness of NABB Metric group contrast 24

Different NAABs may represent different functionality 25

Robustness of NABB Metric x UPDRS associations 27

Supplementary References 29

Detailed description of combined PD volunteer samples

Supplementary Table 1 Detailed descriptions of the two PD volunteer samples combined for this study.

	Zokai et al. (2021)[1]	Heideman et al. (2020)[2]
N	12	16
Groups matched in	Age<br>Education	Age<br>Education
Inclusion Criteria	Currently not participation in ongoing drug trial<br><br>Not taking any of the following drugs:<br>Psychotropic hypertensive<br>Vasoactive medication<br>Long-acting dopamine agonists<br><br>No history of neurological or psychiatry disorders other than PD.<br><br>Tolerating coming off dopaminergic medication.	Being diagnosed with PD within the last 5 years.<br><br>Being able to understand written and spoken instructions in English.<br><br>Being older than 50 years.<br><br>Not taking long-acting dopamine agonists.<br><br>Tolerating coming off dopaminergic medication.
Dopaminergic Medication	Off-Medication<br>Since 7 p.m. of previous day.	Off-Medication<br>Since 7pm of previous day.
UPDRS administration	By trained clinician.	By trained clinician.
Ethics-Reference	12/SC/0650	12/SC/0650
Cohort	Participants were recruited from neurological clinics in Oxfordshire (UK).	Dementias and Neurodegeneration Speciality (https://dendron.org.uk)
Sex (female/male)	8/4	7/9
Handedness (right/left)	11/1	14/2
Age (years) (mean, range)	68.5 (57-77)	68.5 (54-79)
Education (years) (mean, range)	16 (10-24)	13.69 (10-23)
Years since Diagnosis (years) (mean, range)	3 (1-7)	2.75 (1-4)
UPDRS III motor score (mean, range)	32.33 (20-55)	28.86 (11-49)
Hoehn and Yahr scale (mean, range)	1.75 (1-3)	1.78 (1-3)
Levodopa-equivalent daily dose (mg/day) (mean, range)	283.75 (150-540)	427.34 (0-900)*

N = number of participants; UPDRS = Unified Parkinson's Disease Rating Scale

* Two volunteers with PD did not take Levodopa (or equivalent) medication.

Detailed description of demographic and clinical variables

Supplementary Table 2 Demographical and clinical details of included volunteers.

Dataset	Group	Sex	Age	Handedness	Years Since Diagnosis	ACE/ MoCA	H&Y	UPDRS-III	LED
NZ	HC	M	71	R	n.a.	98[a]	n.a.	n.a.	n.a.
NZ	HC	M	69	R	n.a.	100 [a]	n.a.	n.a.	n.a.
NZ	HC	F	69	R	n.a.	99 [a]	n.a.	n.a.	n.a.
NZ	HC	F	63	R	n.a.	99 [a]	n.a.	n.a.	n.a.
NZ	HC	M	71	R	n.a.	n.a. [a]	n.a.	n.a.	n.a.
NZ	HC	M	70	R	n.a.	100 [a]	n.a.	n.a.	n.a.
NZ	HC	F	68	R	n.a.	93 [a]	n.a.	n.a.	n.a.
NZ	HC	M	71	R	n.a.	86 [a]	n.a.	n.a.	n.a.
NZ	HC	M	75	R	n.a.	97 [a]	n.a.	n.a.	n.a.
NZ	HC	M	62	R	n.a.	94 [a]	n.a.	n.a.	n.a.
NZ	HC	F	80	R	n.a.	n.a.	n.a.	n.a.	n.a.
NZ	HC	M	66	R	n.a.	98 [a]	n.a.	n.a.	n.a.
NZ	HC	F	71	R	n.a.	96 [a]	n.a.	n.a.	n.a.
NZ	HC	F	66	R	n.a.	100 [a]	n.a.	n.a.	n.a.
NZ	HC	F	65	R	n.a.	100 [a]	n.a.	n.a.	n.a.
NZ	HC	M	69	R	n.a.	n.a.	n.a.	n.a.	n.a.
NZ	HC	M	75	R	n.a.	99 [a]	n.a.	n.a.	n.a.
NZ	HC	M	69	R	n.a.	n.a.	n.a.	n.a.	n.a.
SH	HC	F	63	R	n.a.	n.a. [b]	n.a.	n.a.	n.a.
SH	HC	M	70	R	n.a.	n.a. [b]	n.a.	n.a.	n.a.
SH	HC	M	74	R	n.a.	n.a. [b]	n.a.	n.a.	n.a.
SH	HC	M	67	L	n.a.	n.a. [b]	n.a.	n.a.	n.a.

SH	HC	M	68	R	n.a.	n.a. [b]	n.a.	n.a.	n.a.
SH	HC	M	69	R	n.a.	n.a. [b]	n.a.	n.a.	n.a.
SH	HC	n.a.	67	R	n.a.	n.a. [b]	n.a.	n.a.	n.a.
SH	HC	F	72	R	n.a.	n.a. [b]	n.a.	n.a.	n.a.
SH	HC	M	60	R	n.a.	n.a. [b]	n.a.	n.a.	n.a.
SH	HC	F	62	R	n.a.	n.a. [b]	n.a.	n.a.	n.a.
SH	HC	M	66	R	n.a.	n.a. [b]	n.a.	n.a.	n.a.
SH	HC	M	60	R	n.a.	n.a. [b]	n.a.	n.a.	n.a.
SH	HC	M	68	R	n.a.	n.a. [b]	n.a.	n.a.	n.a.
SH	HC	M	74	R	n.a.	n.a. [b]	n.a.	n.a.	n.a.
SH	HC	M	76	R	n.a.	n.a. [b]	n.a.	n.a.	n.a.
SH	HC	M	63	R	n.a.	n.a. [b]	n.a.	n.a.	n.a.
SH	HC	F	64	R	n.a.	n.a. [b]	n.a.	n.a.	n.a.
SH	HC	F	68	R	n.a.	n.a. [b]	n.a.	n.a.	n.a.
SH	HC	F	67	L	n.a.	n.a. [b]	n.a.	n.a.	n.a.
NZ	PD	F	57	R	1	99 [a]	1	35	150
NZ	PD	F	70	R	4	99 [a]	1	30	300
NZ	PD	M	69	R	1	91 [a]	1	55	540
NZ	PD	M	69	R	2	98 [a]	1.5	41	300
NZ	PD	F	69	L	5	96 [a]	1.5	22	150
NZ	PD	F	61	R	4	97 [a]	2	29	150
NZ	PD	F	71	R	2	94 [a]	3	29	240
NZ	PD	F	69	R	2	100 [a]	2	31	400
NZ	PD	F	77	R	2	94 [a]	1	32	150
NZ	PD	F	74	R	5	97 [a]	2	20	400
NZ	PD	M	68	R	1	96 [a]	3	36	350

NZ	PD	M	68	R	7	n.a.	2	28	275
SH	PD	M	68	R	2	n.a. [b]	3	49	400
SH	PD	M	63	R	1	n.a. [b]	2	23	300
SH	PD	F	68	R	2	n.a. [b]	1	43	400
SH	PD	M	73	R	2	n.a. [b]	2	48	300
SH	PD	F	54	R	2	n.a. [b]	1	14	n.a.*
SH	PD	M	59	L	2	n.a. [b]	1.5	17	400
SH	PD	F	72	R	1	n.a. [b]	1.5	30	300
SH	PD	M	62	R	4	n.a. [b]	2.5	37	380
SH	PD	F	74	R	4	n.a. [b]	2	27	900
SH	PD	F	73	R	4	n.a. [b]	2	23	887.5
SH	PD	F	74	R	4	n.a. [b]	2	27	900
SH	PD	M	65	R	4	n.a. [b]	1	11	600
SH	PD	M	75	L	2	n.a. [b]	1	19	n.a.*
SH	PD	M	66	R	2	n.a. [b]	2	38	320
SH	PD	F	79	R	4	n.a. [b]	2	21	300
SH	PD	M	71	R	4	n.a. [b]	2	35	450

H&Y = Hoehn & Yahr Stage; UPDRS = Unified Parkinson Disease Rating Scale; ACE = Addenbrookes Cognitive Examination; MoCA = Montreal Cognitive Assessment; n.a. = not available; HC = Healthy Controle; PD = Parkinson's Disease volunteer;
NZ = Zokaei et al.[1]; SH = Heidemann et al.[2];
[a] ACE-scores; [b] MoCA-scores acquired in Heideman et al. were not available.
* Two volunteers with PD did not take Levodopa (or equivalent) medication.

Free Energy of different TDE-HMM fits

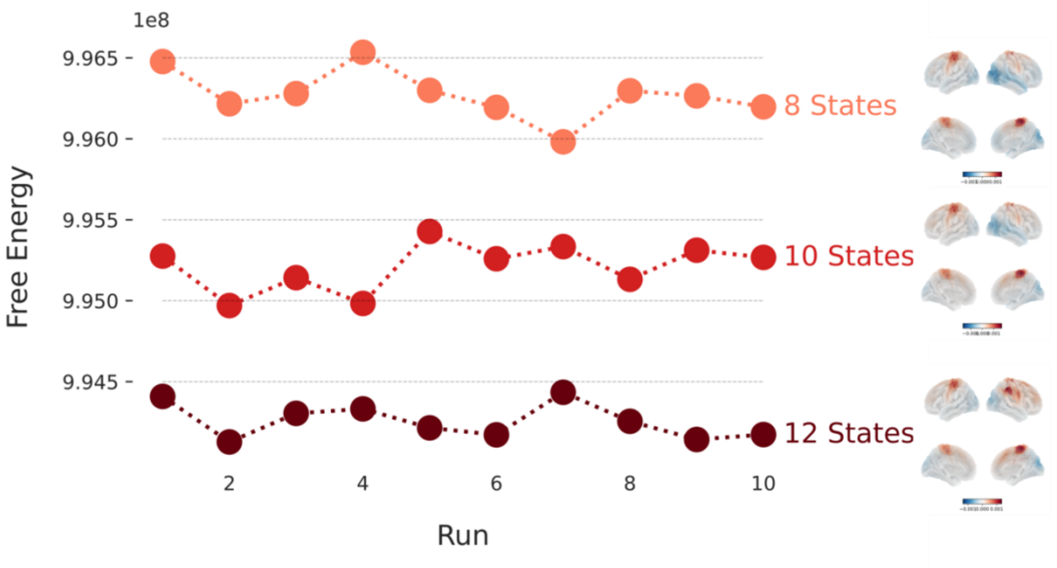

Supplementary Figure 1 Free Energy values of all HMM fits. Free energy values for each of the 30 HMM fits are represented by a dot. Colours mark HMMs inferring different numbers of states. Wideband power maps of the sensorimotor network for the HMM fit with the lowest free energy of all HMMs extracting the same number of states are presented on the right.

Supplementary Figures for GLM-Analyses

Summary of GLMs used for group comparisons between HCs and PD patients.

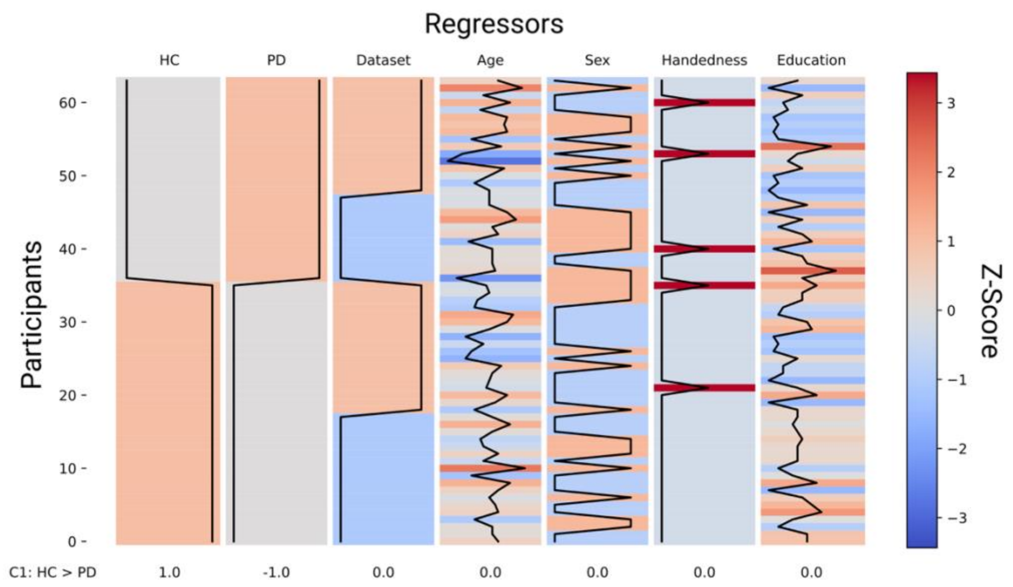

Supplementary Figure 2 Summary of GLMs used to compare variables between HCs and PD groups. Regressors added to GLMs are represented as bars, with each row representing the z-scored regressor value of a single participant. The design matrix for the contrast between HCs and PD groups (C1) is depicted below. Note that the contrast is specified in such a manner that positive t-statistics indicate larger values for participants with PD and negative t-statistics indicate larger values for HCs.

Summary of GLMs used to associate Variables with Motor Symptom Severity Scores.

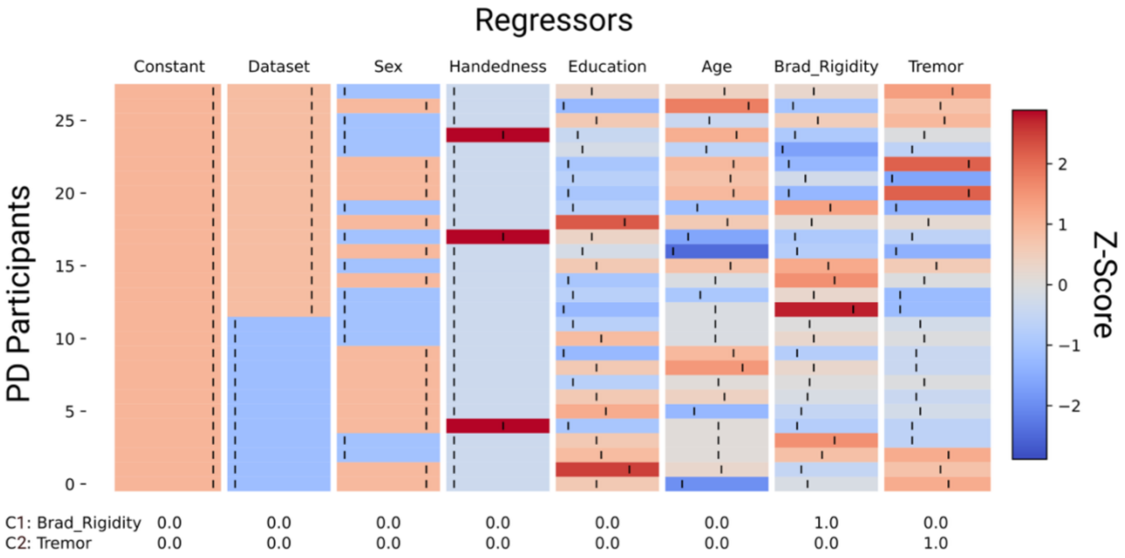

Supplementary Figure 3 Summary of GLMs used to calculate associations between variables and motor symptom severity scores. Bars represent regressors included in the GLMs, with each row corresponding to the z-scored regressor value of a single participant. The design matrix for testing associations between the dependent variable and Bradykinesia/Rigidity or Tremor scores is presented below.

TDE-HMM State Overview

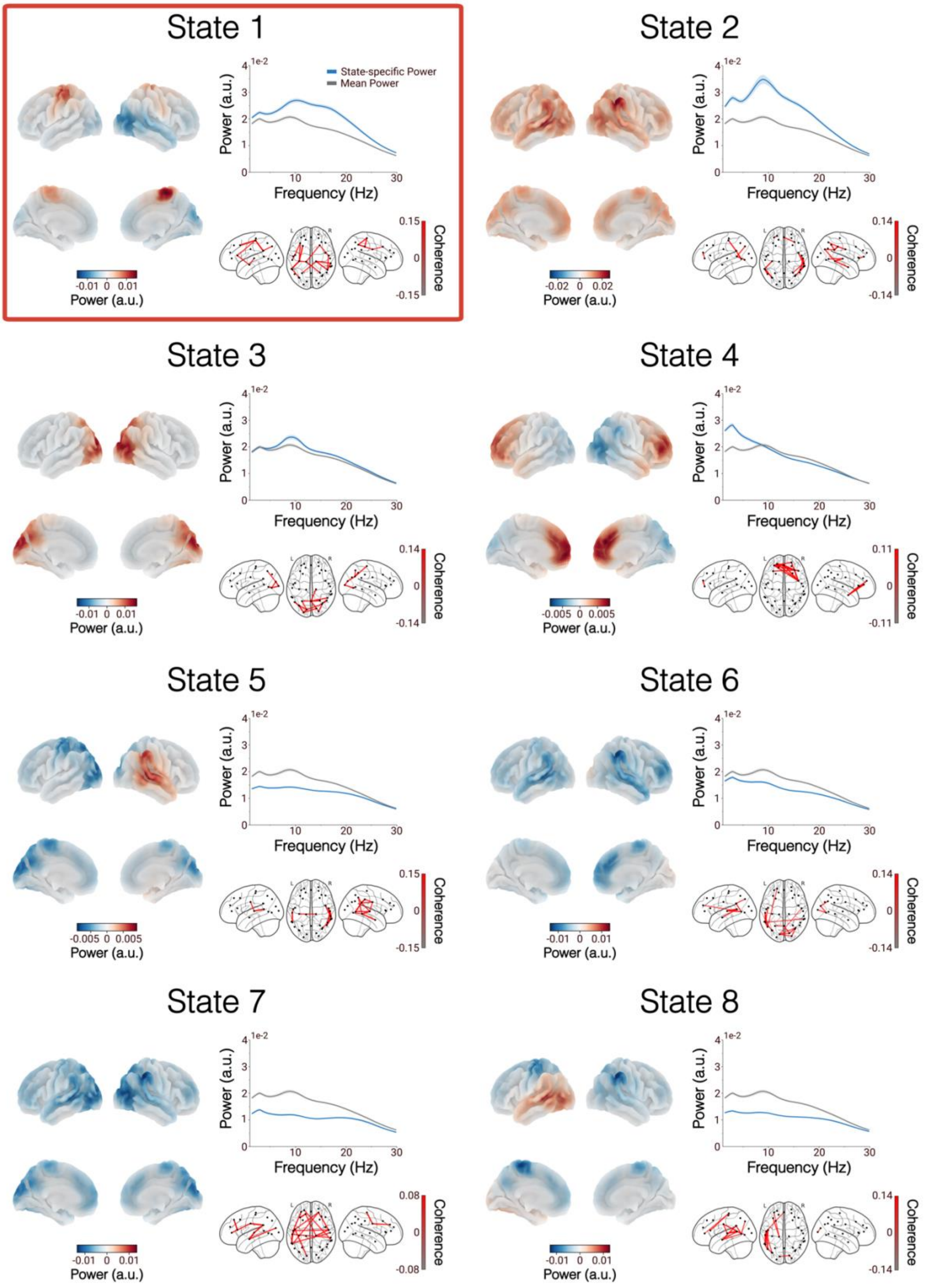

Supplementary Figure 4. Dynamically switching large-scale networks inferred with TDE-HMM. State 1, corresponding to the sensorimotor network, is highlighted in red. Each state is depicted using three panels that present averaged data across all participants. Left: Spatial maps of oscillatory power (2–30 Hz), shown relative to the mean power across all states, projected onto cortical surfaces. Top right: State-specific motor cortical power spectra (blue) compared to the time-averaged power spectrum across all states (grey). Bottom right: Coherence networks (2–20 Hz) thresholded at the 97th percentile to highlight prominent connections.

Spatial correlations between TDE-HMM states and established sensorimotor network

To quantify the spatial correspondence of activations of State 1 and the conventionally described sensorimotor network, we calculated spatial correlations between state-specific wideband power maps (2 to 30 Hz) and a template sensorimotor network extracted from functional imaging (fMRI and PET) activation maps of nearly 30,000 participants.[3] Resting-state network maps were downloaded from FSL (https://www.fmrib.ox.ac.uk/datasets/brainmap+rsns/) and resampled with FLIRT[4,5] to match voxel dimensions of the 8-mm MNI152 standard brain template that was used for source reconstruction of the MEG data. Subsequently, the resampled map of the template sensorimotor network was parcellated into the same weighted parcellation that was used on the MEG data, and spatial correlations between HMM state-specific power maps and the sensorimotor network were calculated for each participant using Pearson's correlation.

Results from this correlation analysis (Supplementary Figure 5) demonstrated strong correlations between the template sensorimotor network and HMM-State 1 (mean = 0.75, SD = 0.06), while all the other states showed weaker spatial correlations. This finding provides evidence that HMM-State 1 reflects the sensorimotor network.

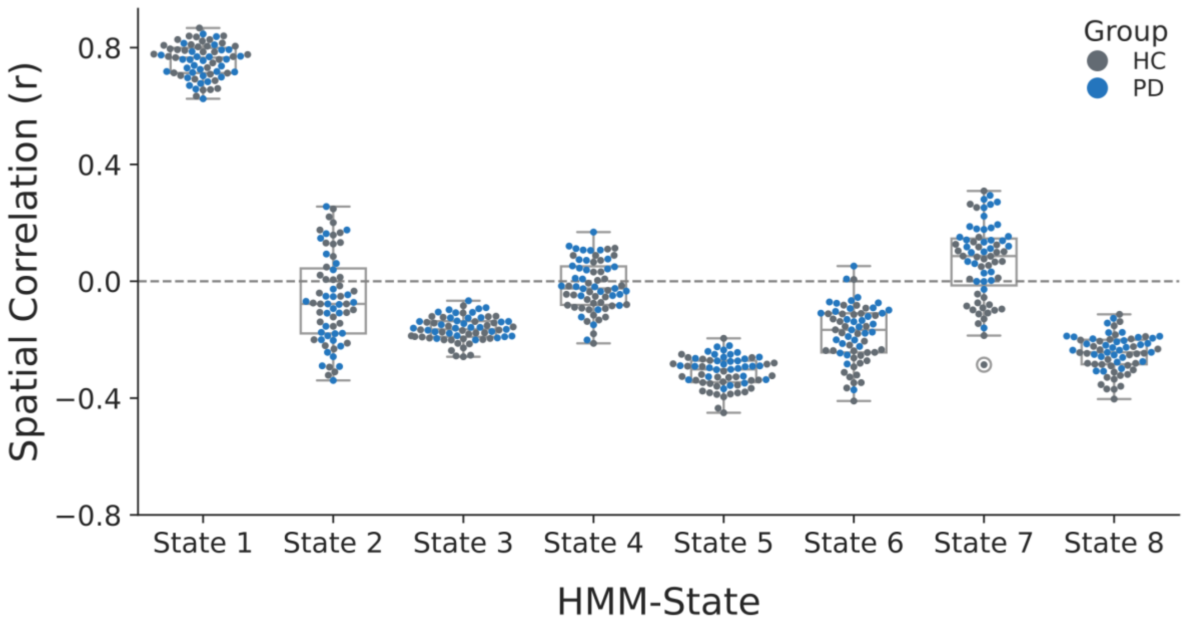

Supplementary Figure 5 Spatial correlations between HMM state-specific wideband power maps and a template sensorimotor network. State-specific wideband power maps were computed for each participant and correlated with a canonical sensorimotor network.[3] Each dot represents the spatial correlation between the template network and a specific HMM state's power map for one participant. Blue dots correspond to Parkinson's disease (PD) participants, and grey dots to healthy controls (HCs). Only HMM-State 1 shows consistently strong positive correlations across all participants (mean r = 0.75, SD = 0.06), providing evidence that HMM-State 1 corresponds to the sensorimotor network.

HMM-State Metric Analysis

State Metrics across all participants

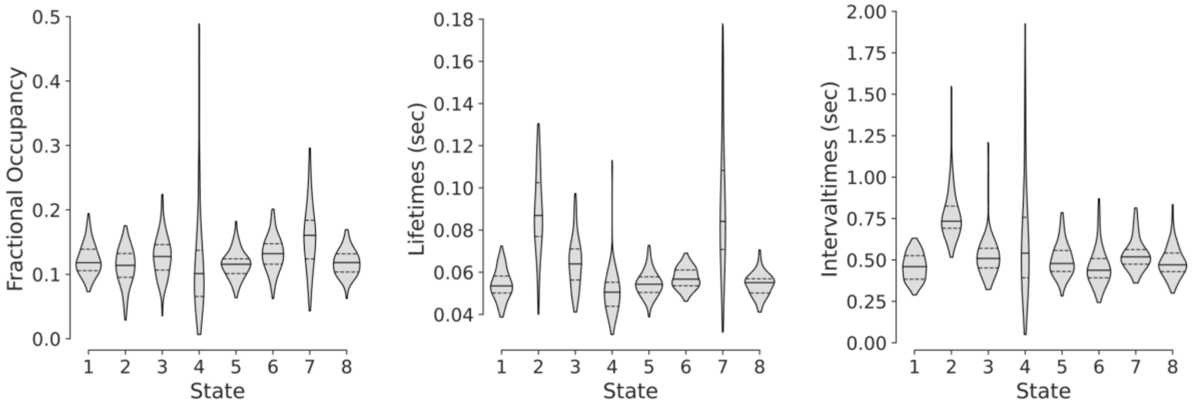

Supplementary Figure 6 State Metrics of TDE-HMM fit with the lowest free energy, extracting 8 states. Qualitative observations show that all fractional occupancies are between .03 and 0.47, indicating that the extracted HMM-states mix well within and between participants.

State Metric Group Contrast

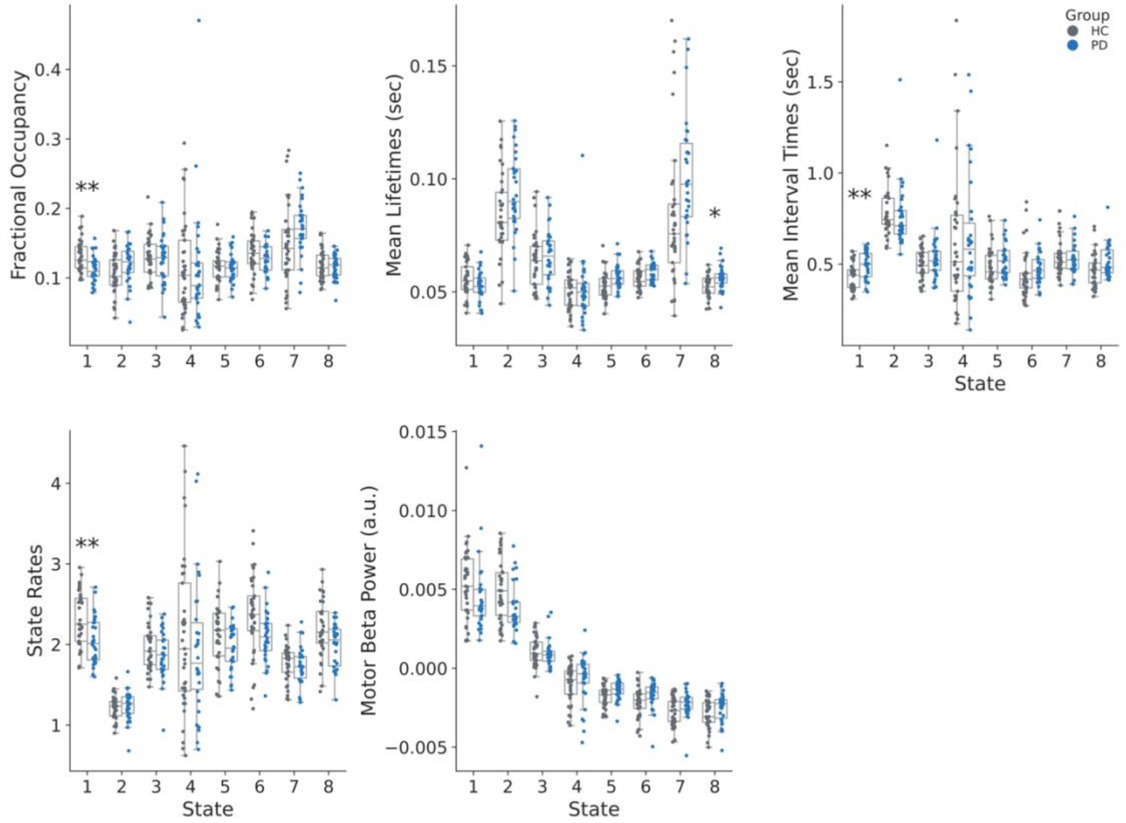

Supplementary Figure 7 Large-scale network dynamics group contrast between HCs and PD patients. Fractional occupancies, mean lifetimes, mean interval times, state rates, and motor beta power change of HCs (grey) and PD patients (blue). The significance of group differences is assessed with maximum t-statistic permutation tests, controlling for multiple comparisons across states. T-statistics were calculated from GLMs accounting for confounds. State metrics of large-scale networks whose occurrence results in a decrease of beta power are plotted in shaded colours.
** indicates $p < .01$ and * $p < .05$.

HMM-State Metric Analyses Robustness

State Matching Procedure

Analyses calculating TDE-HMM state metric group contrasts and state metric x UPDRS score associations were repeated across all 30 HMMs to check whether key findings in the reported HMM fit generalise across different initialisations and number of states. Since the initialisation of the TDE-HMM is random, the order of inferred states is arbitrary, and states have to be matched before assessing the robustness of the observed effects. To match states across different runs inferring the same number of states, we first identified the HMM with the lowest free energy and matched the states of all the remaining HMMs to it. To match states between 2 HMMs, observation models for all states were extracted, and the correlation distance between all states of the 2 HMM runs was calculated. This resulted in several states x number of states distance matrix. The Hungarian algorithm was applied to these distance matrices to find an optimal state assignment between the states of the 2 HMMs with a minimal overall distance. This procedure was performed separately for all HMMs inferring 8, 10 or 12 states. To also allow for comparisons between runs with different numbers of states, the states of the HMMs with the lowest free energy, extracting 10 and 12 states, were matched to the HMM with the lowest free energy, inferring 8 states. Correlation distances were calculated between all states of the HMMs, resulting in an 8x10 or 8x12 distance matrix. These distance matrices were zero-padded to a square shape of either 10x10 or 12x12 so that the Hungarian algorithm could be applied to them. States of the HMMs with 10 or 12 states that were allocated to one of the states of the 8-state HMM were carried on for the robustness check, whereas states matched to one of the zero-padded columns were discarded. States of the remaining HMMs, inferring 10 or 12 states with higher free energy, were first matched to the HMM with the lowest free energy, inferring the same number of states, before being matched to the HMM with the lowest free energy, inferring 8 states.

Robustness of Group Contrast

Due to the stochastic initialisation, the HMM fitting routine may yield slightly different solutions across runs. We next demonstrate that our results are consistent despite this variability and not a fluke from a single run of the HMM fitting routine. We qualitatively assessed whether group contrasts of state metrics from 30 HMM initialisations with lower free energy (poorer model fit) and varying numbers of states showed trends consistent with those from the best-fitting 8-state HMM that is analysed in the main text. This approach allowed us to visualise the sampling distribution of key hypothesis tests in the paper and to demonstrate that the reported test statistics fall well within this distribution. This provides a qualitative assessment of the accuracy associated with the test statistics reported in the main text.

To this end, GLMs (Supplementary Figures 2 & 3) were repeated for key analyses reported in the main text across all model initialisations. The 5th to 95th percentile range of the resulting *t*-statistics was then computed to assess variability across initialisations. Instances where the t-statistics from the best-fitting 8-state HMM lie outside this percentile range are considered not robust across initialisations and selected numbers of states. This approach was chosen over formal significance testing, as the aim was to assess the consistency of the overall trend across models, rather than significance within each individual (potentially poorer fitting) HMM.

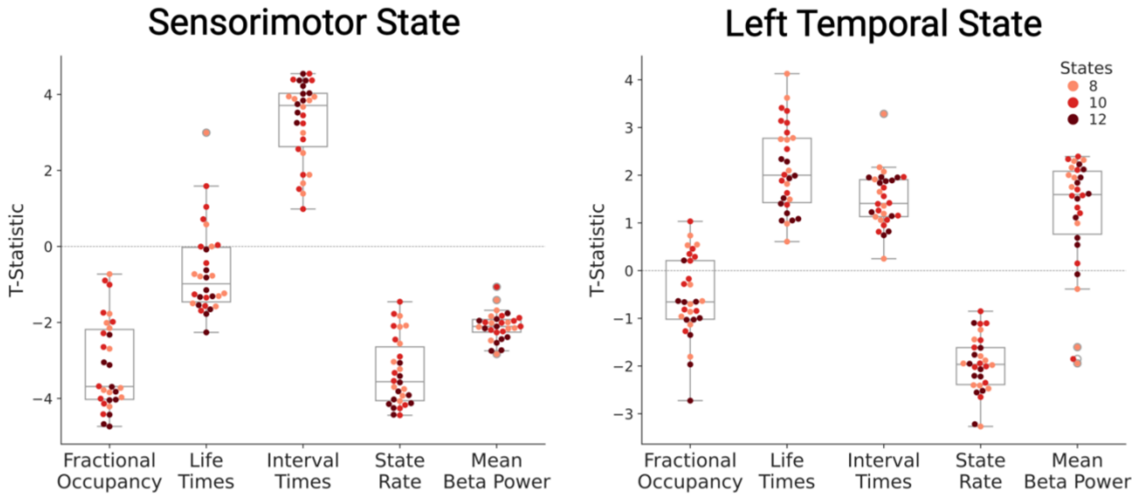

Supplementary Figure 8 Group differences of sensorimotor network metrics are robust across all HMM inferences. GLMs assessing group contrasts (Supplementary Figure 2) of state metrics for the sensorimotor and left temporal states were calculated across 30 HMM initialisations with lower free energy and varying numbers of states. Each dot represents the t-statistic for a group contrast between HCs and PD patients from a single HMM, coloured by the number of inferred states. The 5th to 95th percentile ranges of t-statistic distributions for sensorimotor metrics, i.e., fractional occupancy ([-4.56, -0.95]), interval time ([1.44, 4.47]), and state rate ([-4.35, -1.8]), all included the t-statistics ($t_{fractional\ occupancy}$ = -3.97; $t_{interval\ times}$ = 3.84; $t_{state\ rates}$ = -3.69) of the best-fitting 8-state HMM reported in the main text. For the left temporal state's lifetime, the 5th to 95th percentile range ([1.01, 3.53]) also included t-statistics ($t_{lifetime}$ = 2.78) obtained from the best-fitting 8-state HMM.

Robustness of State Metric x UPDRS score association

Associations between Bradykinesia/Rigidity scores and state metrics of the sensorimotor state were calculated for all 30 HMMs. For both states, t-statistics of all associations were smaller than two and closely distributed around 0, indicating that the non-significant associations

between Bradykinesia/Rigidity scores and state metrics were robust across all HMM fits (Supplementary Figure 9).

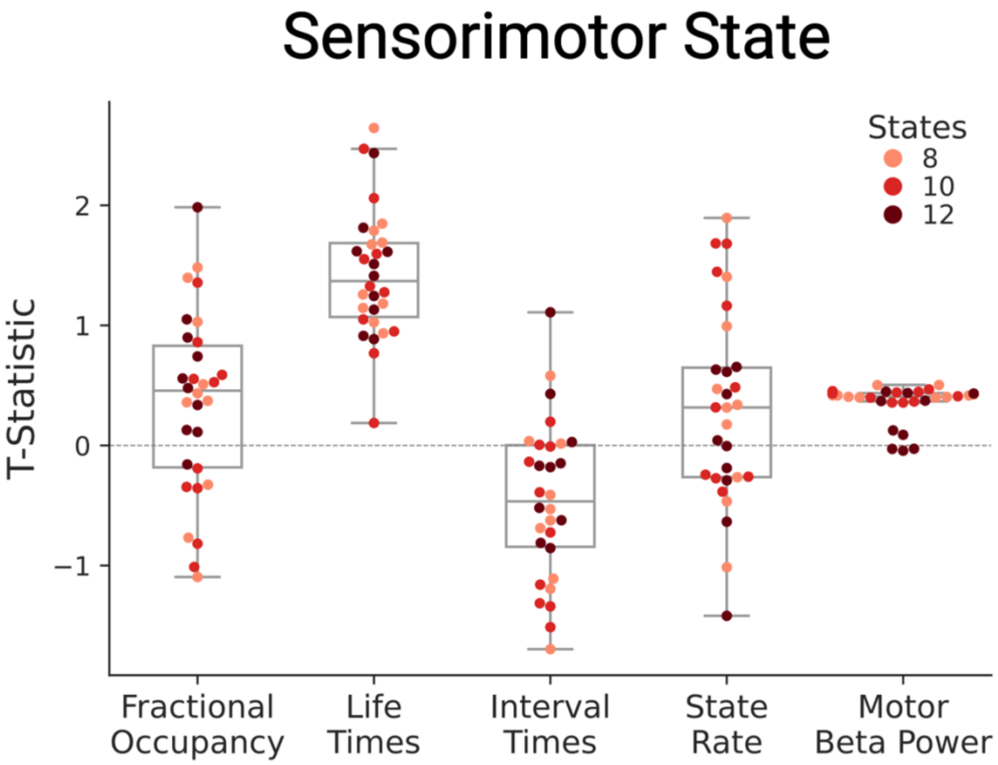

Supplementary Figure 9 Lack of associations between sensorimotor network metrics and Bradykinesia/Rigidity scores is robust across all HMM inferences. GLMs assessing associations (Supplementary Figure 3) between sensorimotor network metrics and Bradykinesia/Rigidity scores were calculated across 30 HMM initialisations with lower free energy and varying state numbers. Each dot represents the t-statistic for the association between Bradykinesia/Rigidity scores and a sensorimotor network metric from a single HMM, coloured by the number of inferred states. The 5th to 95th percentile ranges of t-statistic distributions for fractional occupancy ([-0.92, 1.44]), lifetimes ([0.82, 2.45]), interval times ([-1.44, 0.51]), state rates ([-0.84, 1.68]), and state-specific motor beta power ([-0.03, 0.49]) all include t-statistics

obtained from the best-fitting 8-state HMM ($t_{fractional\ occupancy}$ = 1.03, $t_{lifetimes}$ = 1.79, $t_{interval\ \mathrm{times}}$ = 0.02; $t_{state\ rates}$ = -0.26, $t_{motor\ \mathrm{beta\ power}}$ = 0.42).

Sensorimotor NAABs analysis robustness

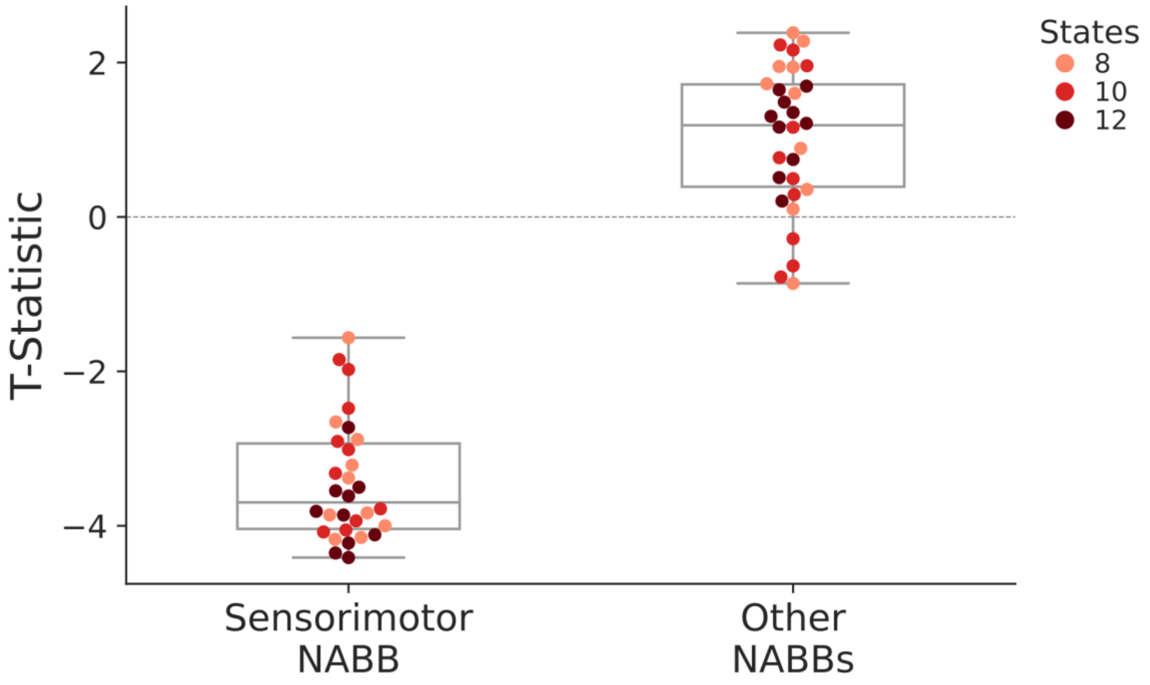

Supplementary Figure 10 Group differences of sensorimotor NABB fractional occupancies between HCs and PD patients are robust across different HMM-inferences and number of states. GLMs assessing group contrasts (Supplementary Figure 2) for sensorimotor NABB and other NABBs fractional occupancies were calculated across 30 HMM initialisations with lower free energy and varying numbers of states. Each dot represents the t-statistic for a group contrast between HCs and PD patients from a single HMM, coloured by the number of inferred states. The 5th to 95th percentile range of t-statistic distributions for sensorimotor NABB fractional occupancy ([-4.29, -1.9]) included the t-statistic obtained from the best-fitting 8-state HMM ($t_{\mathrm{fractional\ occupancy}}$ = -3.98). The 5th to 95th percentile range of the t-

statistic distribution for other NAAB fractional occupancy ([-0.71, 2.26]) did not include the t-statistic from the best-fitting 8-state HMM ($t_{\text{fractional occupancy}} = 2.39$).

NABB Metric Analysis

To better understand whether PD-related increases in the other NABBs analyses were due to balanced increases in all but the sensorimotor NABBs or due to increases in specific NABBs, we calculated NABB metrics for each of the obtained large-scale networks.

We observed that co-occurrences between motor cortical beta bursts and State 7 were significantly more likely in PD patients ($t(58) = 3.01$, $p = .02$) and occurred for significantly longer intervals ($t(58) = -2.98$, $p = .03$) (Supplementary Figure 11). The fact that this increased probability in co-occurrences was correlated with PD patient's Bradykinesia/Rigidity scores ($t(58) = 2.2$, $p = .04$) and high beta coherence ($r = .39$, $p = .002$) suggests that informing the beta burst analysis with TDE-HMM large-scale network dynamics might reveal beta bursts with different functionalities (Supplementary Figure 13). Repeating the NABB analyses for all 30 TDE-HMM fits (10 fits for 8, 10, and 12 states, respectively) demonstrated that the observed group differences in the sensorimotor NABB metrics and State 7 NABB fractional occupancy are robust across different HMM inferences and number of states (Supplementary Figure 12). In contrast, PD-related increases in State 7 NABB lifetimes were not reliably observed across different HMM fits. Significant correlations between State 7 NABBs fractional occupancies and lifetimes and symptom severity scores were robust across all these models (Supplementary Figure 14).

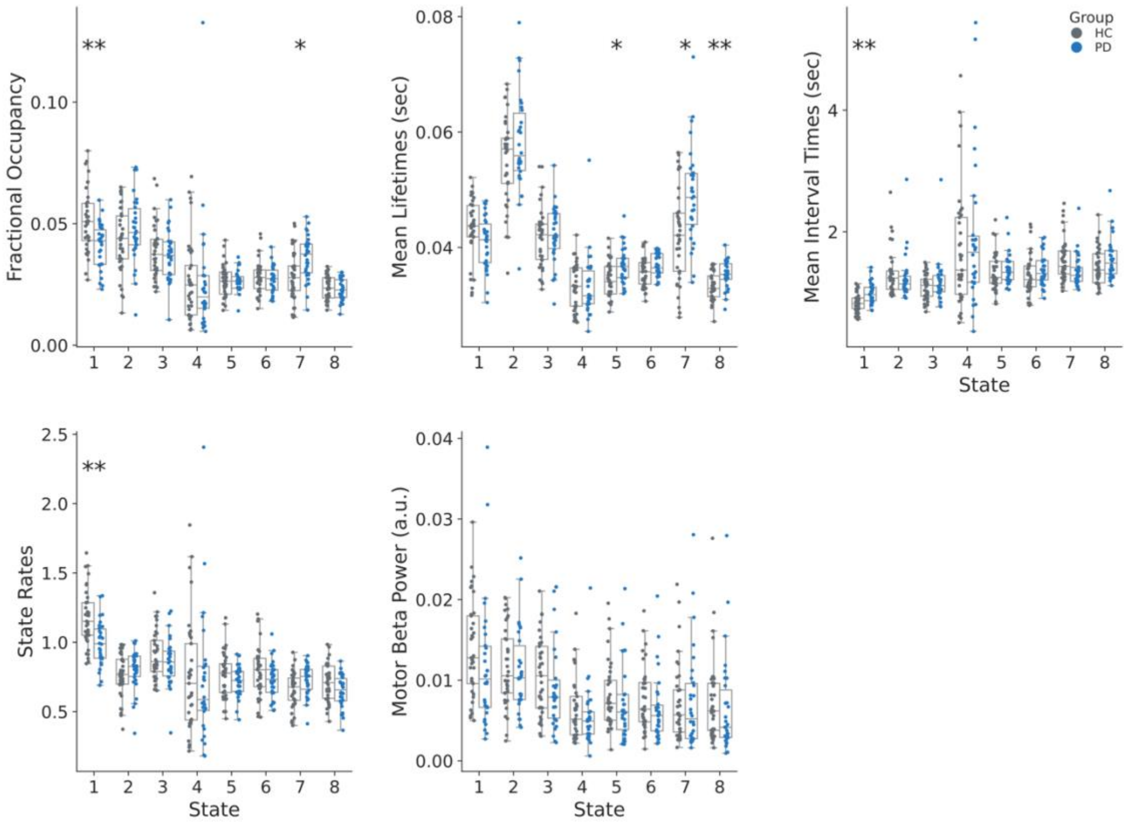

Supplementary Figure 11 Group comparison of network-associated beta burst dynamics (NABBs) between HCs and PD patients. Fractional occupancies, mean lifetimes, mean interval times, state rates, and motor beta power values of HCs (grey) and PD patients (blue) are depicted. The significance of group differences is assessed with maximum t-statistic permutation tests controlling for multiple comparisons across states. T-statistics were calculated from GLMs accounting for confounds. State metrics of NABBs of large-scale networks that have decreased motor cortical beta power are plotted in shaded colours. ** indicate $p < .01$ and * $p < .05$.

Robustness of NABB Metric group contrast

Robustness checks of group contrasts of metrics of the State 7 state and sensorimotor NABBs demonstrated that the above-reported significant differences are robust across all HMM fits (Supplementary Figure 12).

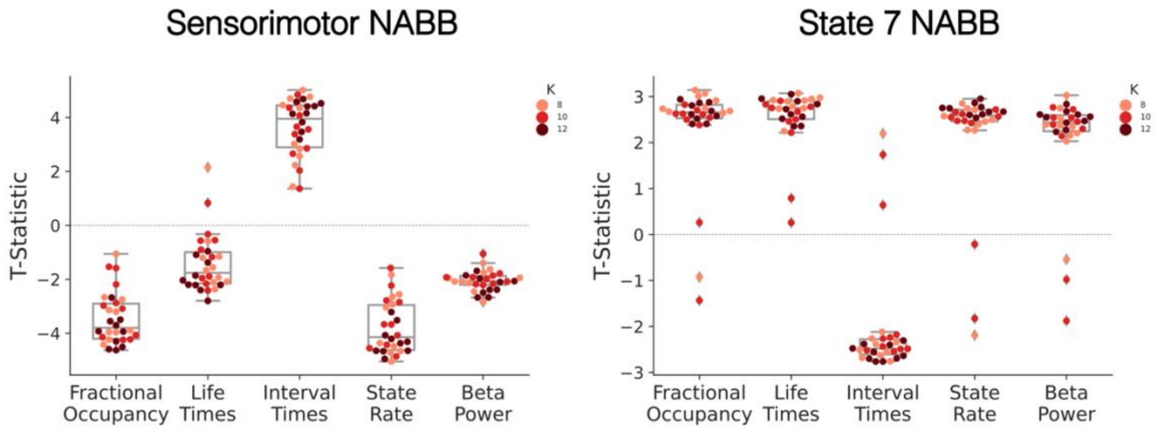

Supplementary Figure 12 Group differences of sensorimotor and State 7 network-informed burst metrics are robust across all HMM inferences. GLMs assessing group contrasts (Supplementary Figure 2) of state metrics for the sensorimotor and State 7 NABBs were calculated across 30 HMM initialisations with lower free energy and varying numbers of states. Each dot represents the t-statistic for a group contrast between HCs and PD patients from a single HMM, coloured by the number of inferred states. The 5th to 95th percentile ranges of t-statistic distributions for sensorimotor NABB fractional occupancy ([-4.55, -1.55]), interval times ([1.71, 4.81]), state rates ([-4.91, -2.01]), State 7 NABB fractional occupancy ([-0.39, 3.04]), and State 7 NABB lifetimes ([1.43, 3.02]) all included t-statistics obtained from the best-fitting 8-state HMM (sensorimotor NABB: $t_{\text{fractional occupancy}} = -3.98$, $t_{\text{interval times}} = 4.33$, $t_{\text{state rates}} = -4.41$; State 7 NABB: $t_{\text{fractional occupancy}} = 2.95$, $t_{\text{lifetimes}} = 3.08$).

Different NAABs may represent different functionality

To check whether the extraction of NABBs may have the potential to disentangle beta events with different functionalities, we assessed associations between NABB fractional occupancies and other variables such as Bradykinesia/Rigidity scores and motor cortical beta power and coherence. Differing association patterns may provide evidence for a dissociation of the NABB functionality.

The analyses found significant associations between the sensorimotor NABBs' fractional occupancies and beta power but not beta coherence and Bradykinesia/Rigidity scores. On the other hand, no significant relationships between State 7 NABBs' fractional occupancies and beta power were found, whereas motor cortical beta coherence and Bradykinesia/Rigidity scores were significantly linked to State 7 NAAB fractional occupancies (Supplementary Figure 13).

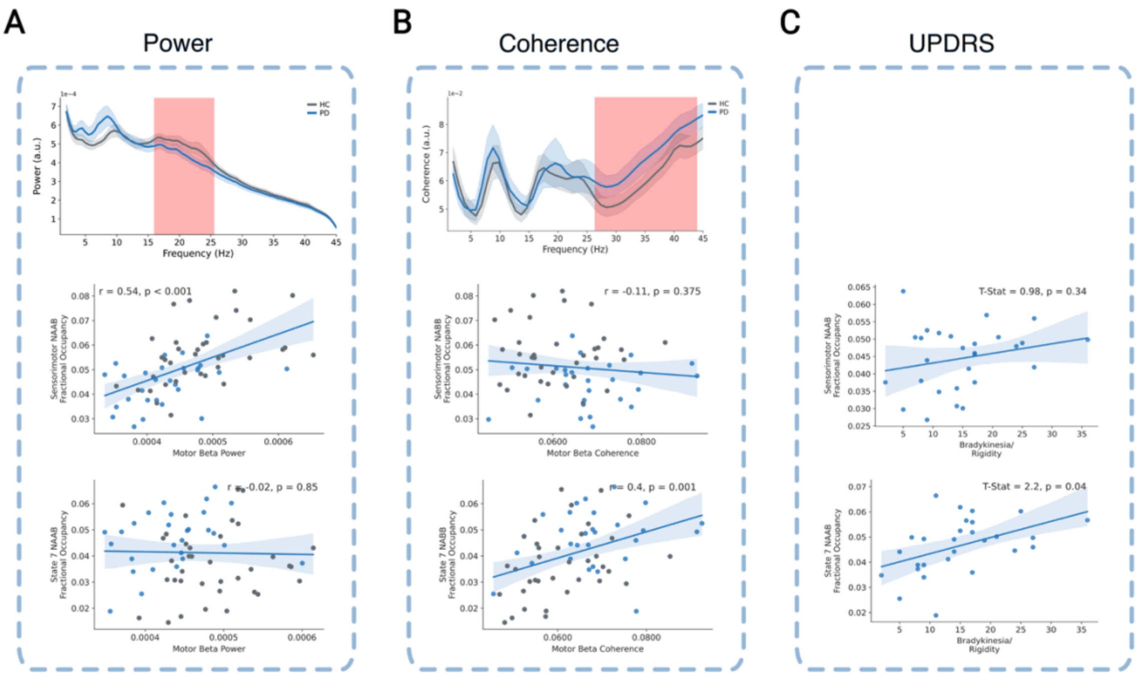

Supplementary Figure 13 Sensorimotor and State 7 network-associated beta bursts show distinct associations with beta power, beta coherence, and Bradykinesia/Rigidity scores. **A** Top: Motor cortical group-averaged power spectra for HC (grey) and PD groups (blue). A significant 16-25.5 Hz cluster (peak = 22.6 Hz; mean $t(57) = 2.65$, p = .03), identified via GLMS (Supplementary Figure 2) and cluster-based permutation testing, is marked in red. Middle: Positive Pearson's correlation between 16-25 Hz motor power and Sensorimotor NABB fractional occupancy ($r(62) = .54$, $p < .001$). Bottom: No significant Pearson's correlation between 16-25 Hz motor power and State 7 NABB occupancy ($r(62) = -.002$, $p = .85$).

B Top: Motor cortical-cortical group-averaged coherence spectra for HC (grey) and PD groups (blue). A significant 26-44 Hz cluster (peak = 33.2 Hz; mean $t(57) = -2.89$, $p = .002$), via GLMs (Supplementary Figure 2) and cluster-based permutation testing, is marked in red. Middle: No significant Pearson's correlation between coherence and Sensorimotor NABB occupancy ($r(62) = -.11$, $p = .375$). Bottom: Positive Pearson's correlation between coherence and State 7 NABB fractional occupancy ($r(62) = .4$, $p = .001$).

C Associations between Bradykinesia/Rigidity scores and fractional occupancy assessed with GLMs (Supplementary Figure 3). Middle: No significant relationship between Bradykinesia/Rigidity scores and Sensorimotor NABB fractional occupancy ($t(12) = 0.98$, $p = .34$). Bottom: Significant positive association between Bradykinesia/Rigidity scores and State 7 NABB fractional occupancy ($t(12) = 2.2$, $p = .04$). Each dot represents a participant; HCs are shown in grey, PD individuals in blue.

These differing association patterns hint at different functionalities of the State 7 and sensorimotor NAABs, with changes in the State 7 NAAB relevant to motor symptom severity in PD. The wideband power profiles of State 7 indicate a brain-wide decrease in 2 to 20Hz

power (Figure 3) while high beta to gamma motor cortical-cortical coherence is increased (Supplementary Figure 13). Since this is an exploratory post-hoc analysis, these findings should be treated carefully, and further replication in other studies is required.

Robustness of NABB Metric x UPDRS associations

Robustness checks of the association between Bradykinesia/Rigidity scores and metrics of the State 7 and sensorimotor NABBs demonstrate that associations between Bradykinesia/Rigidity scores and State 7 NABBs' fractional occupancies and lifetimes are robust across HMM fits. No significant associations between sensorimotor NABBs and Bradykinesia/Rigidity scores were found across all HMM fits (Supplementary Figure 14).

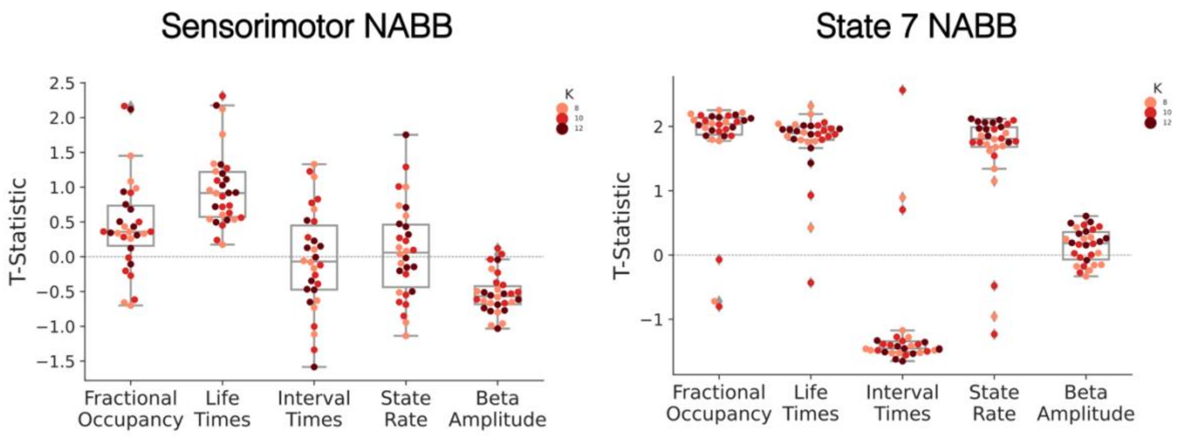

Supplementary Figure 14 Associations between State 7 network-associated beta burst metrics and Bradykinesia/Rigidity scores are robust across all HMM inferences. GLMs assessing associations (Supplementary Figure 3) between Bradykinesia/Rigidity scores and sensorimotor or State 7 NABB metrics were calculated across 30 HMM initialisations with lower free energy and varying state numbers. Each dot represents the t-statistic from a single HMM, coloured by the number of inferred states. The 5th to 95th percentile range of the t-statistic distribution for the association between Bradykinesia/Rigidity scores and State 7

NABB fractional occupancy ([-0.19, 2.17]) includes the t-statistic obtained from the best-fitting 8-state HMM reported in the main text ($t_{\text{fractional occupancy}} = 2.14$).

Supplementary References

1. Zokaei N, Quinn AJ, Hu MT, Husain M, van Ede F, Nobre AC. Reduced cortico-muscular beta coupling in Parkinson's disease predicts motor impairment. *Brain Communications*. 2021;3(3):fcab179. doi:10.1093/braincomms/fcab179

2. Heideman SG, Quinn AJ, Woolrich MW, van Ede F, Nobre AC. Dissecting beta-state changes during timed movement preparation in Parkinson's disease. *Progress in Neurobiology*. 2020;184:101731. doi:10.1016/j.pneurobio.2019.101731

3. Smith SM, Fox PT, Miller KL, et al. Correspondence of the brain's functional architecture during activation and rest. *Proc Natl Acad Sci USA*. 2009;106(31):13040-13045. doi:10.1073/pnas.0905267106

4. Jenkinson M, Smith S. A global optimisation method for robust affine registration of brain images. *Medical Image Analysis*. 2001;5(2):143-156. doi:10.1016/S1361-8415(01)00036-6

5. Jenkinson M, Bannister P, Brady M, Smith S. Improved Optimization for the Robust and Accurate Linear Registration and Motion Correction of Brain Images. *NeuroImage*. 2002;17(2):825-841. doi:10.1006/nimg.2002.1132
